# The role of windows of selection and windows of dominance in the evolution of insecticide resistance in human disease vectors

**DOI:** 10.1111/eva.12897

**Published:** 2019-12-10

**Authors:** Andy South, Rosemary Lees, Gala Garrod, Jessica Carson, David Malone, Ian Hastings

**Affiliations:** ^1^ Liverpool School of Tropical Medicine (LSTM) Liverpool UK; ^2^ Innovative Vector Control Consortium (IVCC) Liverpool UK; ^3^ Present address: Bill & Melinda Gates Foundation London UK

**Keywords:** dose–response, drug resistance, insecticide resistance, insecticide resistance management, malaria, vector‐borne diseases, window of dominance, window of selection

## Abstract

Persistent insecticides sprayed onto house walls, and incorporated into insecticide‐treated bednets, provide long‐acting, cost‐effective control of vector‐borne diseases such as malaria and leishmaniasis. The high concentrations that occur immediately postdeployment may kill both resistant and susceptible insects. However, insecticide concentration, and therefore killing ability, declines in the months after deployment. As concentrations decline, resistant insects start to survive, while susceptible insects are still killed. The period of time after deployment, within which the mortality of resistant individuals is lower than that of susceptible ones, has been termed the “window of selection” in other contexts. It is recognized as driving resistance in bacteria and malaria parasites, both of which are predominantly haploid. We argue that paying more attention to these mortality differences can help understand the evolution of insecticide resistance. Because insects are diploid, resistance encoded by single genes generates heterozygotes. This gives the potential for a narrower “window of dominance,” within the window of selection, where heterozygote mortality is lower than that of susceptible homozygotes. We explore the general properties of windows of selection and dominance in driving resistance. We quantify their likely effect using data from new laboratory experiments and published data from the laboratory and field. These windows can persist months or years after insecticide deployments. Differential mortalities of resistant, susceptible and heterozygous genotypes, after public health deployments, constitute a major challenge to controlling resistance. Greater attention to mortality differences by genotype would inform strategies to reduce the evolution of resistance to existing and new insecticides.

## 1 INTRODUCTION

Seventeen per cent of human infectious diseases are estimated to be transmitted by vectors such as mosquitoes, ticks and fleas (WHO, 2014a). Malaria alone, despite recent declines, killed an estimated 435,000 people in 2017 (World Health Organisation, 2018). Insecticides, used in public health interventions to control vector‐borne diseases, have saved millions of malaria deaths (Bhatt et al., 2015) and averted deaths and morbidity from other infections such as dengue, Zika, lymphatic filariasis, leishmaniasis and Japanese encephalitis. The control of such diseases is threatened by insecticide resistance that is now widespread in many vector species (Gould, Brown, & Kuzma, 2018; Ranson & Lissenden, 2016). Insecticide resistance management (IRM) programmes have been designed and implemented to slow the evolution of resistance (Denholm & Rowland, 1992; Gould et al., 2018; Huijben & Paaijmans, 2017; Roush, 1989; Sternberg & Thomas, 2017). The design of effective, appropriate IRM strategies depends on understanding the forces that drive the spread of insecticide resistance. Most IRM strategies have been developed for agricultural use, adapted for public health and imported into public health programmes (IRAC, 2011; WHO, 2012). One important operational factor in insecticide deployment is that insecticide concentrations decline after application. In agriculture, this decline tends to be rapid, either as a deliberate policy to avoid residual insecticides on human food, or because the insecticide is rapidly washed off crops by rain or degraded by sunlight. Conversely, most public health applications are specifically designed to deploy highly persistent insecticides in order to maximize their long‐term impact and cost‐effectiveness (White, Conteh, Cibulskis, & Ghani, 2011). The key objective of this paper is to explore how long‐term persistence of insecticides, used in many public health applications, is likely to accelerate the evolution of insecticide resistance.

**Figure 1:**
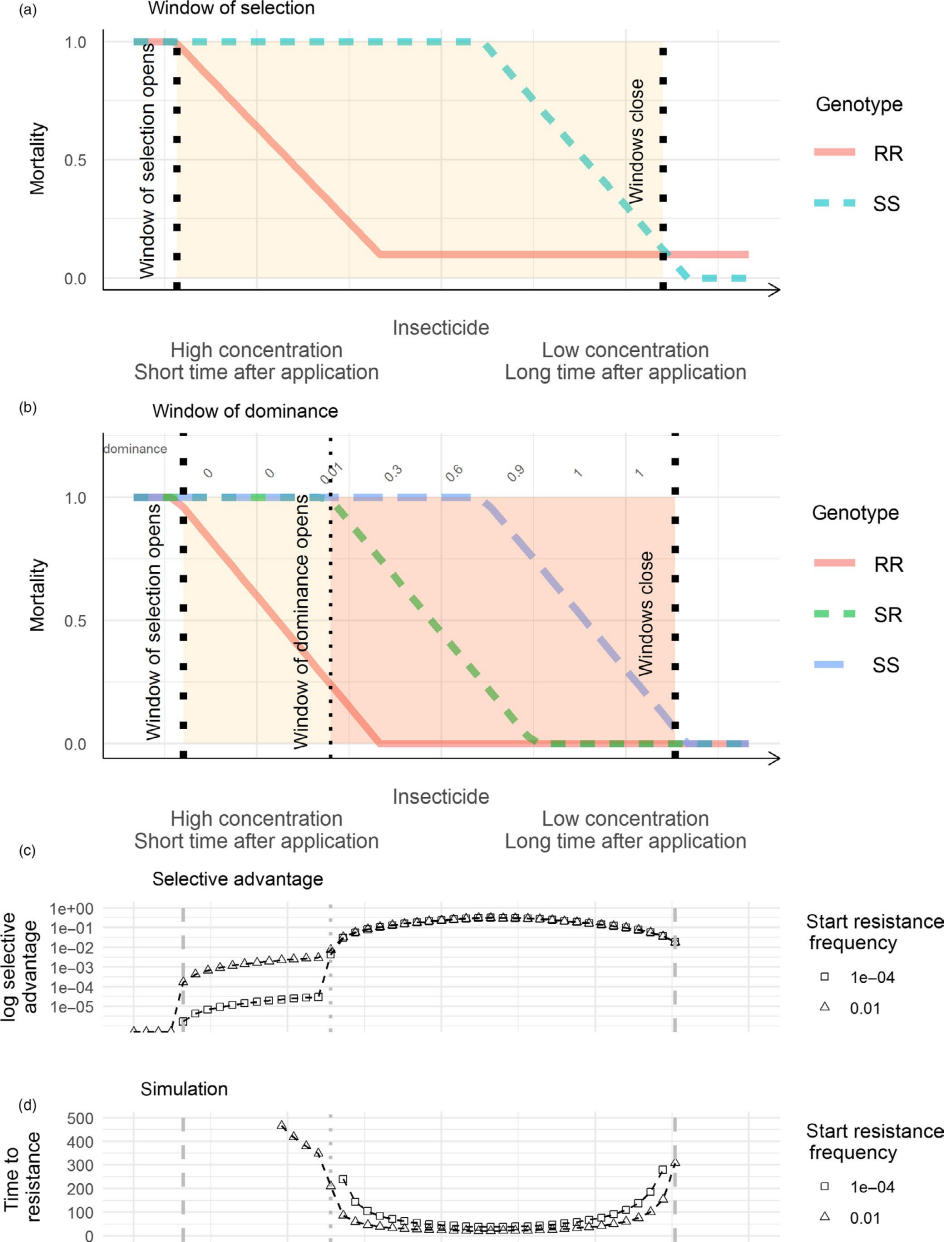
Idealized windows of selection and windows of dominance for insecticide resistance. (a) Window of selection when there are only data on resistant and susceptible strains: selection for resistance occurs when resistant strains have lower mortality than susceptible ones (yellow shaded region). Fitness costs of resistance may cause the mortality of resistant insects to exceed that of susceptible ones at low concentrations, as shown at the lower right, and resistance will be selected against. (b–d) Idealized windows of selection and dominance when resistance is encoded by a single gene, and there are data on mortalities for all three genotypes (SS, SR and RR). The *x*‐axis is shared between (b–d). (b) illustrates how the mortality probabilities change for each genotype, and the row of numbers along the top of the plot is the dominance of resistance at each time point. (c) shows selective advantage per generation which is highest within the window of dominance. (d) shows predicted time until resistance allele frequency reaches 50% for simulations started at each point along the *x*‐axis in (b). Times to the 50% resistance threshold are lowest within the window of dominance, and the threshold of 500 generations is not reached for points outside of the window of selection. (c,d) assume that 30% of all mosquitoes are exposed to the insecticide, equivalent plots at different exposure levels are shown in the Appendix S1–S5

We investigate the impact of insecticide persistence by borrowing the term “window of selection,” which has previously been applied to the evolution of drug resistance in malaria (e.g. Kay & Hastings, 2015; Slater, Okell, & Ghani, 2017) and antibiotic resistance in bacteria (Gullberg et al., 2011). We illustrate the basic principles in Figure 1a. The highest insecticide concentrations occur immediately after application/deployment and, in the best‐case scenario, are sufficient to kill both resistant and susceptible insects. In that case, the window of selection is closed. As concentrations decline, the mortality of resistant insects declines before that of susceptible ones, leading to the differential survival that drives the evolution of resistance; the selective window is open. In many places, resistance to target doses means that the window of selection is already open on deployment. After further decline, insecticide concentrations reach levels such that both resistant and susceptible forms survive, resistance is not selected for, and the window closes again. If there are fitness costs, the mortality of resistant individuals may be higher than that of susceptible individuals at lowest concentrations and this would be expected to select for a return to susceptibility. The window of selection for resistance could be defined in terms of differential fitness, as anything that allows the resistant individuals to leave more progeny will promote selection (such as better mating success; Rowland, 1991). However, for operational purposes it is likely that mortality is the most important and measurable factor.

Previous discussion of windows of selection has compared resistant and susceptible “strains.” In malaria and bacteria, this easily equates to selection on a single gene (or plasmid) because these organisms are haploid when they encounter the drug in humans, so in the simplest cases have only two genotypes, that is R and S. However, insects are diploid with potential for three distinct genotypes at a resistance locus (i.e. RR, SR and SS), making the dynamics of selection more complicated (Figure 1b). As insecticide concentrations decline, the relative mortality of the SR genotype changes, reflecting increasing dominance of the resistance gene (see also Denholm & Rowland, 1992; Gould et al., 2018; Levick, South, & Hastings, 2017; Figure 1). At initial high concentrations, dominance is expected to be low and the mortality of the SR close to the SS. As concentrations decline, dominance is also expected to decline and mortality of the SR becomes closer to the RR. We use the term “window of dominance” to describe the region within the window of selection where dominance is greater than zero. The importance of dominance for the evolution of insecticide resistance has been recognized previously (Bourguet, Genissel, Raymond, & Raymond, 2000; Gould et al., 2018; Levick et al., 2017; Mallet, 1989), but the effect on selection of changing dominance, in response to insecticide concentration, has not been quantified.

## 2 METHODS

### 2.1 Population genetic and computational analyses of windows of selection and dominance

Mortality estimates of all three genotypes (SS, SR and RR), where available, allowed us to quantify the magnitude of selection for resistance using two approaches: firstly, using population genetics to calculate the selective advantage of resistance over a single generation, and secondly, using a published computer simulation (Levick et al., 2017; South & Hastings, 2018) to calculate how rapidly evolution drives resistance allele frequency to 50%.

Both approaches require the proportion of the population exposed to the insecticide and the frequency of the resistance allele. The frequency of the resistance allele alters the proportion of insects in each of the three genotypes, and hence the impact of dominance. We used two illustrative starting resistance allele frequencies 0.01 and 10^−4^ (i.e. 1% and 0.01%). The proportion of the population exposed to the insecticide was set at 30% for both sexes [increasing it gave higher selective advantage and lower times to resistance but qualitatively similar results (Figure S2.1)].

#### 2.1.1 Population genetics to calculate the selective advantage of resistance

Let the fitnesses of the SS, SR and RR genotypes exposed to the insecticide be *W*_ss_, *W*_sr_ and *W*_rr_, respectively. Here, we assume fitness is the proportion of the genotype that survives exposure. Let *p* and *q*(=1 − *p*) be the frequency of resistant and susceptible alleles, respectively. Assuming that there is no selection on those not exposed to the insecticide (i.e. that there are no fitness costs of resistance) and that the genotypes are initially in the Hardy–Weinberg equilibrium (i.e. the frequencies of RR, SR and SS genotypes are *p* ^2^, 2*pq* and *q* ^2^, respectively), then the frequencies of p and q next generation, denoted *p*′ and *q*′, are as follows:

$$p\prime =\frac{x\left[p^{2}w_{\text{RR}}+2pq*0.5*w_{\text{SR}}\right]+\left(1-x\right)\left[p^{2}+2pq*0.5\right]}{\bar{W}}$$

$$q^{\prime }=\frac{x\left[2pq*0.5*w_{\text{SR}}+q^{2}w_{\text{SS}}\right]+\left(1-x\right)\left[2pq*0.5+q^{2}\right]}{\bar{W}}$$

where $\bar{W}$ is a normalizing factor equal to the sum of the numerators. The relative fitness of the R allele is given by *p*′/*p*, but fitness is often broken down to *w* = 1 + *z*, where *z* is the selective advantage (we avoid the conventional symbol, *s*, for selective advantage to avoid confusion with the S allele). The value of *z* can therefore be obtained as.

$$z=\frac{p\prime }{p}-1.$$

We generally present values of *z* (rather than *w*) because changes in its value are more obvious in the plots. Note that z will also depend on the frequency of the resistance allele because this frequency determines the relative frequency of insects in the three genotypes.

#### 2.1.2 Computer simulation to calculate times to resistance thresholds

Computer simulation of the evolution of resistance used a published model (Levick et al., 2017; South & Hastings, 2018). The model represents the genetics of a single randomly mixing population and, as in standard population genetic models, tracks frequencies of alleles and genotypes without tracking demography. Hence, it does not include changes in population size or dispersal. The mortalities of resistant and susceptible genotypes, at different time points, were used to calculate model inputs, namely insecticide effectiveness (mortality of the SS), resistance restoration (the proportion of SS mortality that is prevented by the RR genotype) and dominance. The proportion of the population exposed to the insecticide was set at *x* = 0.3 and initial starting frequencies at 0.01 and 10^−4^, as above. A “time to resistance” was calculated as the number of generations taken to reach a resistance allele frequency of 50%. This illustrates the changing selective pressure at each concentration and is compatible with previous analyses using this metric to quantify the rate of evolution of resistance (Birget & Koella, 2015; Levick et al., 2017; South & Hastings, 2018).

### 2.2 Laboratory experiments quantifying the duration of windows of selection

We conducted two experiments to quantify windows of selection in terms of insecticide concentration ranges and timescales postapplication. Windows of selection were indicated where there was a difference in mortality between resistant and susceptible mosquito strains. Note that, because these experiments used strains rather than known genotypes, they did not allow us to measure windows of dominance. The first experiment exposed resistant and susceptible strains of *Anopheles gambiae* to filter papers impregnated with deltamethrin at a range of concentrations and measured mortality. The second experiment exposed resistant and susceptible strains of *A. gambiae, A. funestus* and *Aedes aegypti* to different surfaces (cement, wood and mud) sprayed with deltamethrin. These surfaces were stored at temperatures and humidity representative of sub‐Saharan Africa. Mortality was measured at regular intervals in the 18 months after spraying. Full methods of both experiments are provided in the Appendix S1.

### 2.3 Literature search for existing data to estimate windows of selection and dominance

We searched the literature for other work reporting differences in mortality between resistant and susceptible strains, or individual RR, SR and SS genotypes, that can be used to quantify windows of selection and dominance. Details of search methods are provided in Appendix S3.

All figures were created in R using ggplot2 and patchwork (Pedersen, 2018; R core Team, 2017; Wickham, 2009).

## 3 RESULTS

### 3.1 Theoretical approaches for estimating evolution of insecticide resistance within windows of selection and dominance

The selective advantage of the resistance allele can be calculated from the mortalities of the three genotypes in our idealized plot, Figure 1b, and is shown in Figure 1c. Selection for resistance starts to occur as declining concentrations allow the window of selection to open. There is then a rapid increase in selective advantage as the window of dominance opens, due to declining mortality of the SR. Selective advantage remains high throughout the window of dominance, only declining to zero once concentrations become sufficiently low that all SS survive the same as RR. These patterns are similar for different resistance frequencies except that lower frequencies give lower selective advantage and a greater increase on entering the window of dominance.

The selective advantage, shown in Figure 1c, operates over a single generation. The same pattern (although inverted) occurs when selection is compounded over generations in the computer simulation to estimate time to resistance. Figure 1d plots the time until resistance allele frequency reaches 50%, a conventional endpoint in many studies of insecticide resistance (e.g. Birget & Koella, 2015). The shortest time values (i.e. most rapid selection) occur in the window of dominance. For the lower resistance starting frequency, the simulation does not reach the resistance threshold within 500 generations when outside of the window of dominance. Time to resistance may be >500 generations as the window starts to open, but falls to 21 generations when selection is greatest.

The magnitudes of the selective advantage and time to resistance are both dependent on the proportion of the population exposed to the insecticide. In Figure 1, an exposure value of 0.3 is used. Settings with higher exposure values had higher selective advantage and lower times to resistance, but a qualitatively similar pattern of greatest selection within the window of dominance (Figure S2.1). In summary, both the population genetics equation and simulations indicate evolution of resistance within the window of selection, and faster evolution within the window of dominance.

### 3.2 Laboratory experiments illustrating windows of selection

**Figure 2:**
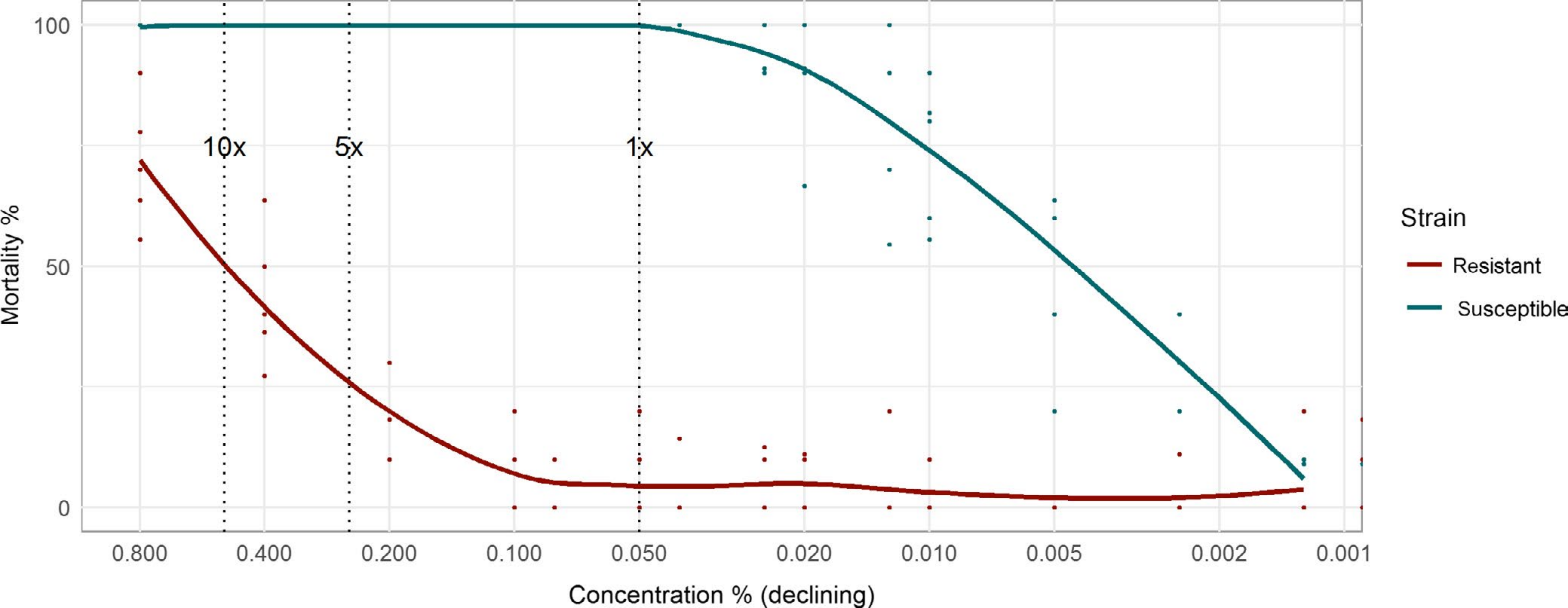
Window of selection in units of insecticide concentration for *Anopheles gambiae* exposed to deltamethrin. Points show the percentage mortality 24 hr after exposure, by replicate, for resistant and susceptible strains, and lines show a locally weighted smoother (loess). The *x*‐axis is plotted from high to low concentrations, for consistency with time plots because concentrations decline with time after deployment. Vertical dashed lines indicate the WHO standard discriminating concentrations at 1×, 5× and 10× used to estimate resistance intensity (WHO, 2013a)

Resistant and susceptible *Anopheles gambiae* strains were exposed to different deltamethrin concentrations (Figure 2). The observed mortality pattern was very similar to the idealized window of selection shown in Figure 1a. At high concentrations, mortality is high for both strains; a window of selection is open at intermediate concentrations where mortality is higher for the susceptible than the resistant strain; and at low concentrations, mortality is low for both strains. The window of selection operates over a 320‐fold range of concentrations, opening around the highest concentration of 0.8% and closing around 0.0025%.

**Figure 3:**
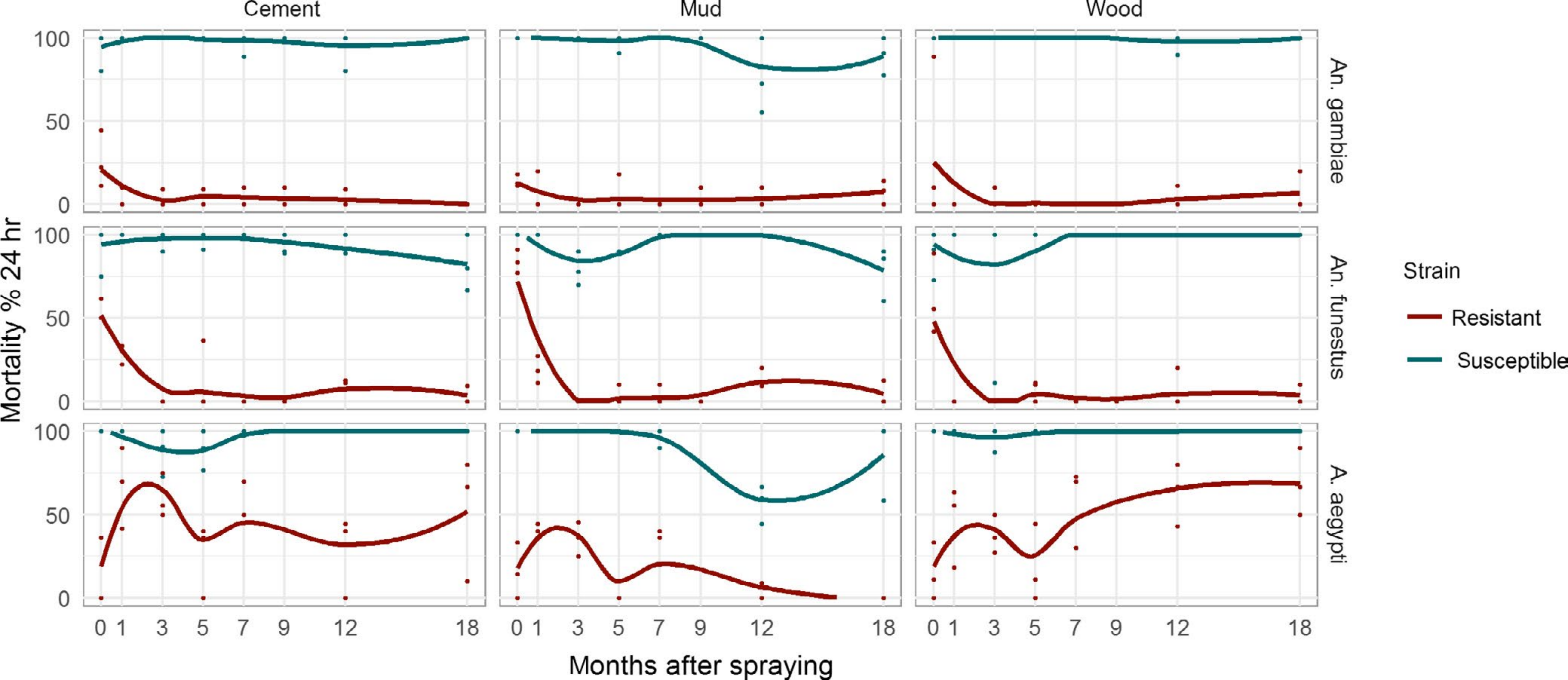
Windows of selection, in units of time, associated with deltamethrin exposure of three vector species: *Anopheles gambiae*, *Anopeheles funestus* and *Aedes aegypti*. Deltamethrin initially applied to three different substrates (cement, mud and wood tiles), then kept in a stability chamber mimicking African field conditions. Percentage mortality was assessed by cone bioassay, 24 hr after exposure, for resistant and susceptible strains. Mortality lines show a locally weighted smoother (loess). Windows of selection are open for all species–surface combinations across the whole time of the experiment. (Control mortality of mosquitoes exposed to tiles not treated with insecticide is shown in Figure S2.3, indicating no difference in resistant and susceptible mortality in the absence of insecticide)

In operational settings, the time (rather than concentration range) for which windows of selection are open is the more important driver of resistance. Figure 3 shows the changing mortality over 18 months, for resistant and susceptible strains of three mosquito species, exposed to three deltamethrin‐sprayed substrates. Large differences, between the mortality of susceptible and resistant strains, can be seen in all combinations of species/substrate, clearly confirming the presence of windows of selection. In all cases, the windows of selection seem to be open immediately after spraying, and not to have closed by month 18. The clearest example is for *An. gambiae* (Figure 3, upper panels), where the window of selection is open at near maximum extent over the full 18‐month period after spraying. The results are equally clear‐cut for *An. funestus* (Figure 3, middle panels), where the window of selection is open for the whole 18‐month period, although maximal differential mortality does not arise until around 3 months post‐treatment. In *Ae. aegypti* (Figure 3, lower panels), the window of selection is open for 18 months, but the difference in mortality between resistant and susceptible strains is slightly less than in the two Anopheline species. The apparent increase in mortality of the resistant strain on wood, between 5 and 18 months after spraying, is counter‐intuitive and probably reflects declining colony health. Nevertheless, resistant mortality remains lower than susceptible, suggesting the window of selection remains open.

### 3.3 Published work illustrating windows of selection

**Figure 4:**
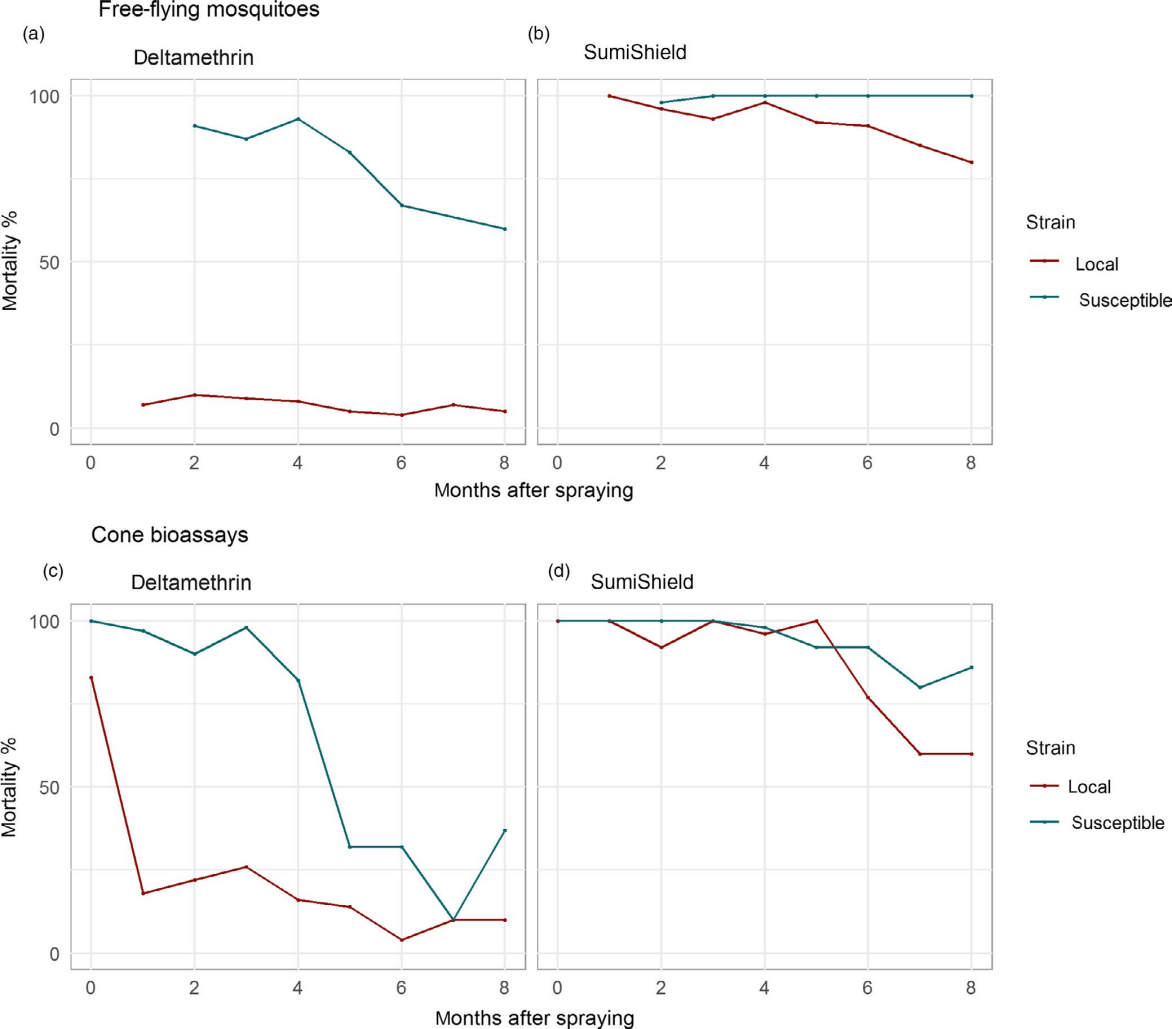
Windows of selection in units of time for *Anopheles* mosquitoes in sprayed experimental hut trials in Benin; data extracted from Figures 3, 4, 5, 6 of Agossa et al. (2018). (a,b) Free‐flying mosquitoes, (c,d) cone bioassays. (a–c) show 24‐hr mortality for deltamethrin, and (b and d) show 120‐hr (5 day) mortality for clothianidin (SumiShield) (a slower acting neonicotinoid insecticide for which 5‐day mortality is a better measure). A window of selection is open from deployment for deltamethrin and starting to close by month 8. Conversely, for clothianidin the window of selection is initially shut and opens in later months

Our laboratory experiments above indicated windows of selection. In Table 1, we summarize other published studies illustrating windows of selection and dominance. These are further described in the Appendix S3, together with replotted data. These published studies confirm the results described above; that is, that windows of selection are routinely observed and that their magnitude is large, often covering several hundred‐fold changes in concentration, and typically persisting for many months or years. As an illustrative example, Figure 4 shows changing mortality of *Anopheles* strains in the months after spraying deltamethrin and clothianidin (SumiShield), replotted from Agossa et al. (2018). For deltamethrin, free‐flying mosquitoes in hut trials (Figure 4a) suggested a window of selection of at least 8 months and those in cone bioassays (Figure 4c) suggested 5 months. For clothianidin, a newer insecticide, there appears to be a window of selection opening 6 months after spraying (Figure 4b,d).

**Table 1:** Durations of windows of selection and dominance measured in this study and from the literature

Paper	Organism	Genetics	Mortality measurement	Time or concentration	Insecticide	Duration^a^ of windows of selection	Duration of windows of dominance	Figure
This study, South et al. (2019)	Anopheles Anopheles & Aedes	Strains	Cone bioassays on filter papers and sprayed surfaces	Concn. Time	Deltamethrin	320× 18 months		2 3
Agossa et al. (2018)	Anopheles	Strains	Free‐flying and cone bioassays in sprayed huts	Time	Deltamethrin Clothianidin	7 months 2 months		4
Anshebo et al. (2014)	Anopheles	Strains	Cone bioassays on treated nets	Time & Concn.	Deltamethrin	>12 months 6.5×		S3.1
Bagi et al. (2015)	Anopheles	Strains	Bottle assays	Concn.	Permethrin	400×		S3.2
Etang et al. (2016)	Anopheles	Strains	Tube assays and cone bioassays	Concn. & # net washes	Deltamethrin treated nets	100× 35 washes		S3.3
Mahama, Desiree, Pierre, and Fabrice (2007)	Anopheles	Strains	Cone bioassays on treated nets	Time	Deltamethrin treated nets	12 months		S3.4
Li et al. (2008)	Ticks	Genotypes	Larval bioassay	Concn.	Permethrin	450×	15X	5
Georghiou and Taylor (1986)	Culex mosquitoes	Genotypes	Larval bioassay	Concn.	Permethrin	1400×	85X	6
Corbel et al. (2004)	Anopheles	Genotypes	Tunnel test	Concn.	Permethrin treated nets	5× (the entire range tested)	5X	S3.5
McKenzie and Whitten (1982)	Blowfly	Genotypes	Larvae exposed on sheep	Time	Dieldrin Diazinon	30 weeks 19 weeks	30 weeks 11 weeks	S3.6

a Duration of windows of selection (WoS) and dominance (WoD) is given as approximate x‐fold difference in concentration or in units of time (i.e. concentration on closing divided by concentration on opening). Note that WoD can only be determined if mortality is by genotypes.

**Figure 5:**
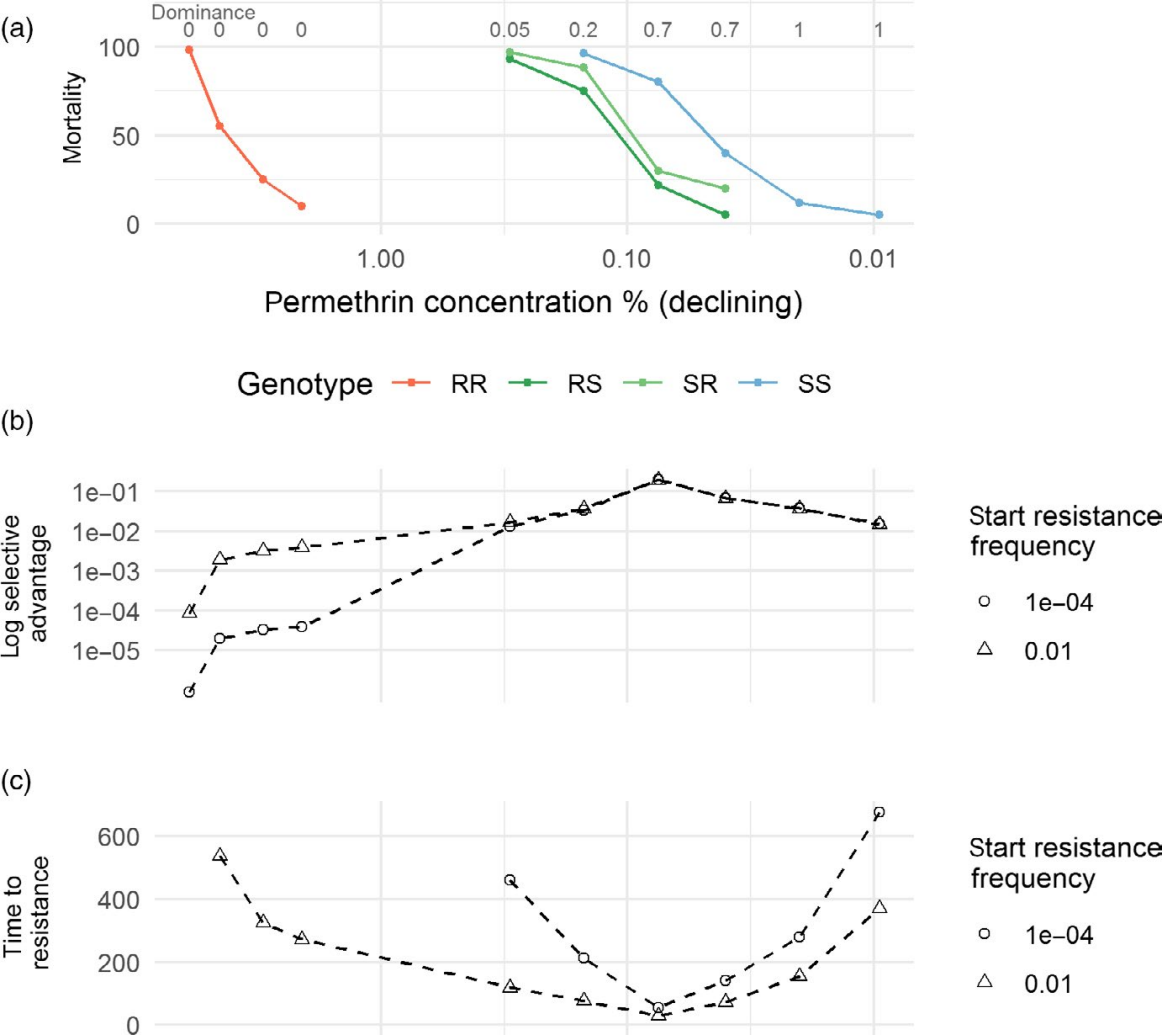
Windows of selection and dominance, in units of concentration, associated with permethrin resistance in the tick species *Boophilus microplus*; (a) shows the mortality data reported in Figure 1 of Li et al. (2008) from bioassays on tick larvae of a susceptible, resistant and F1 crosses exposed for 24 hr. The row of numbers along the top of (a) is our calculation of dominance of resistance at each concentration. (b and c) show our measures of selection plotted along the same concentration *x*‐axis. Where mortality data were absent, we extrapolated to 0 or 100% to extract values for the calculation (e.g. mortality of RR was assumed to be 0 at concentrations ≤1%, and mortality of RS and SR was assumed to be 100% at concentrations >0.5%). We used the mean mortality of the two heterozygous genotypes (in the original experiment, the SR came from SS fathers and RR mothers, RS from the reverse). Panel b shows how selective advantage within a single generation changes during the windows of selection and how it depends on the starting frequency of resistance. (c) shows simulation results of the number of generations needed to reach a resistance allele frequency of 50%

**Figure 6:**
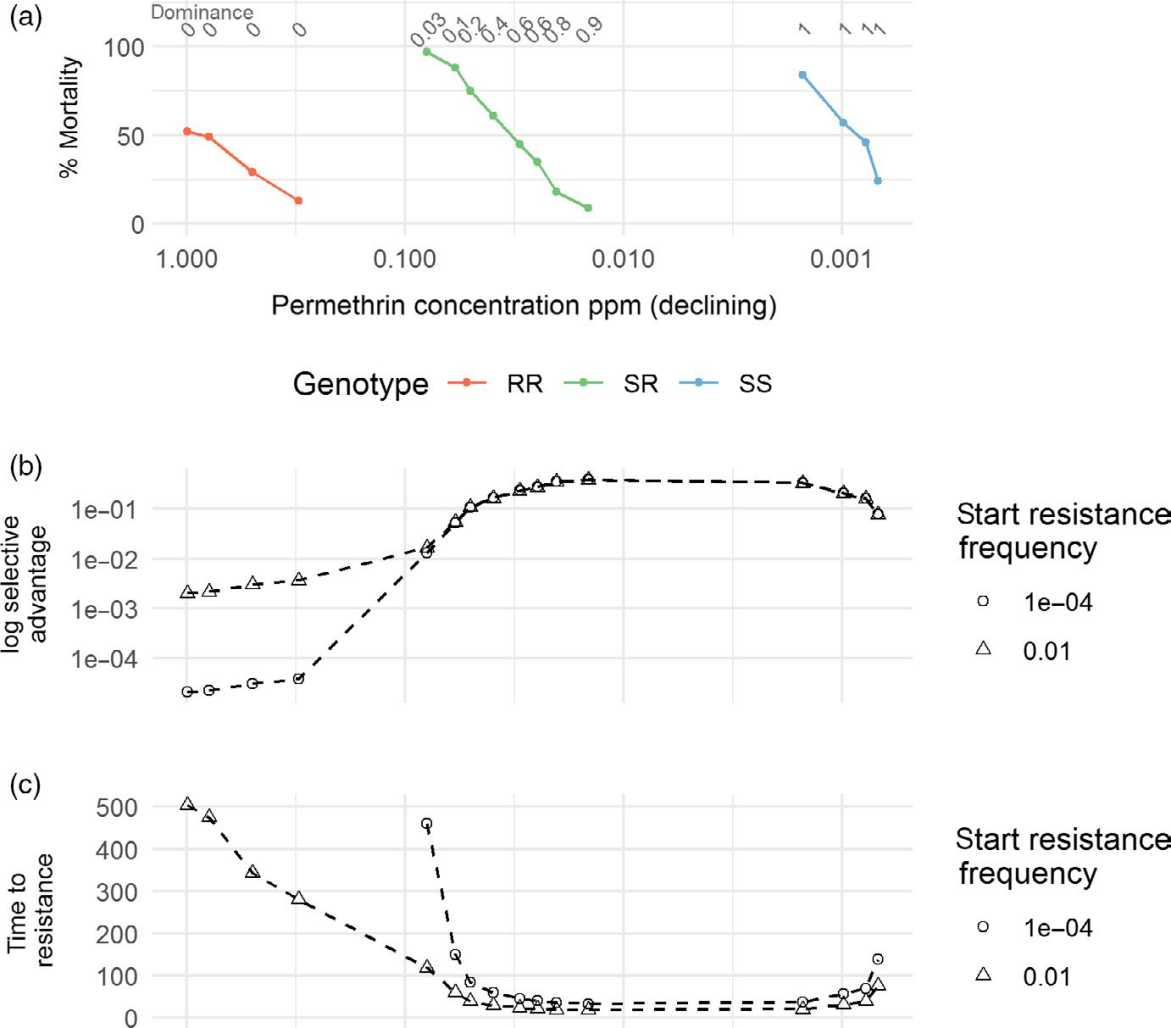
Windows of selection and dominance, in units of concentration, associated with permethrin resistance in *Culex quinquefasciatus*. (a) shows data reported in Georghiou and Taylor (1986) from larvae exposed to the insecticide. The row of numbers along the top of (a) is our calculation of dominance of resistance for each concentration. (b and c) show our measures of selection calculated on the same concentration *x*‐axis. Where mortality data were absent, we extrapolated to 0 or 100% to extract values for the calculation (e.g. mortality of RR was assumed to be 0 at concentrations <0.1 ppm, and mortality of SS was assumed to be 100% at concentrations >0.01ppm). (b) shows how selective advantage changes during the windows of selection and how it depends on the starting frequency of resistance. (c) shows simulation results of the number of generations needed to reach a resistance allele frequency of 50%

Windows of dominance can be estimated from rarer published studies reporting mortalities of RR, SR and SS genotypes exposed to a range of insecticide concentrations or times after deployment (Table 1). Windows of dominance, where SR mortality is less than SS, are observed in all studies, but exhibit qualitatively different patterns. In one example, with cattle ticks, the window of dominance occurs over a narrow range because the mortalities of SR and SS decline together and are close to each other (Figure 5a). In another example, with Culex mosquitoes, the window of dominance is relatively wide as a result of a greater concentration gap between the SR and SS mortality curves (Figure 6a). The theoretical predictions from these study data are similar to those from the idealized example shown in Figure 1b,c; that is, selective advantage and time to resistance (e.g. panels b & c of Figures 5 and 6) are most intense during the window of dominance. This is particularly true for lower starting frequencies of resistance. The changes in the measures of selection vary according to the mortality patterns described above. For the ticks (Figure 5), there is a single peak in selection at a relatively low concentration, due to the SR mortality not declining until low concentrations. A longer period of high selective advantage is shown for Culex (Figure 6), because the window of dominance is open over a wider concentration range.

**Figure 7:**
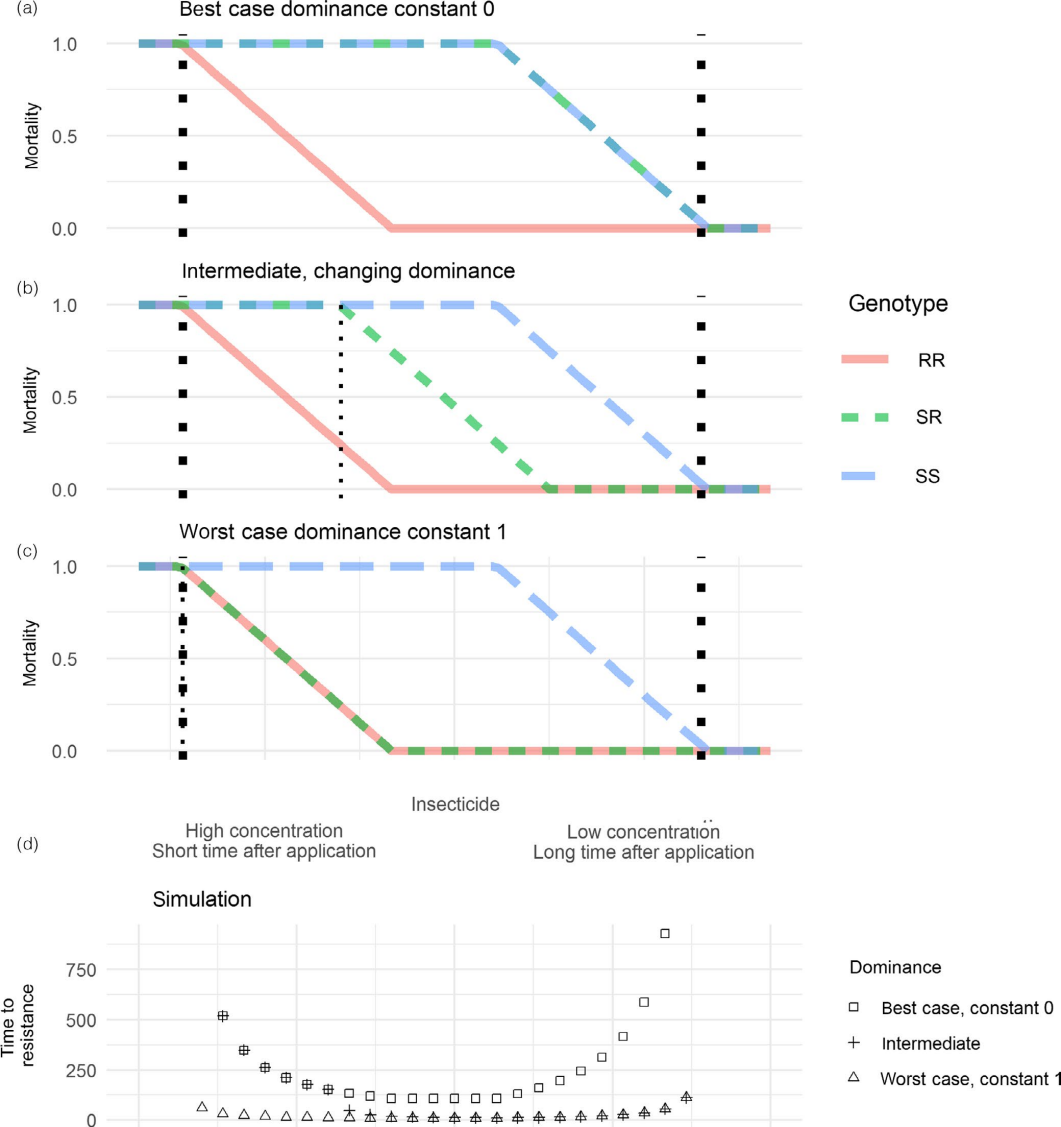
Implications for selection of not knowing dominance values. (a–c) show three scenarios for a window of selection. (a) a best case where dominance is constant 0, mortality of the SR is the same as the SS, and there is no window of dominance. (b) an intermediate scenario the same as Figure 1b. (c) a worst‐case scenario where dominance is a constant 1, and the window of dominance is open for the whole of the window of selection. The *x*‐axis is shared between a–d. (d) shows predicted time until resistance allele frequency reaches 50% for simulations started at each point along the *x*‐axis in panels a–c. The difference between the best‐ and worst‐case scenarios can be hundreds of generations

These results show the importance of dominance, and that the precise nature of mortality differences between genotypes, with concentration, is needed to know the implications for selection. Often, those data are not available. To illustrate how different selection can be under different dominance values, we ran our simulation model under three scenarios: a best case with dominance constant at zero; a worst case with dominance constant at 1; and an intermediate case the same as our idealized example in Figure 1b. The results showed that uncertainty in dominance values, at either high or low concentrations, could lead to differences in predictions of times to resistance of hundreds of generations (Figure 7).

## 4 DISCUSSION

Windows of selection and dominance are straightforward ideas that emerge from three simple principles: firstly, that the mortality of resistant strains is lower than susceptible ones, when exposed to some insecticide concentrations; secondly, that insecticide efficacy declines with decreasing concentration, or its surrogate, time since deployment; and thirdly, that dominance of resistance genes is not a fixed genetic parameter, but is likely to increase as concentrations decline. These principles combine to create a window of selection, in units of time or concentration, where insecticide resistance is selected, and within which a window of dominance occurs where selection is much stronger. We provide idealized representations of these windows of selection and dominance in Figure 1. We show, using data generated by ourselves (Figures 2 and 3) and others (Figures 4, 5, 6 and Appendix S3), that this idealized representation matches observed field and laboratory data.

The window of selection in our specific example of deltamethrin and *An. gambiae* extended over about a 320‐fold concentration range (Figure 2). Operationally, it is the length of time these windows are open and the patterns of differential mortality within the window that are the critical factors driving insecticide resistance. Our sprayed surface experiment shows that these differences, between resistant and susceptible mortality, can last for more than 18 months (Figure 3). Published data, for spray and nets, suggest windows of selection can act over wide concentration ranges and be open for months or years (Table 1).

The most accessible examples of windows of selection are obtained by comparing resistant and susceptible strains, over a range of concentrations or times postdeployment. The reasoning is that the resistant and susceptible strains can represent the range of genotypes potentially present in a local population. The problem with this comparison is that it is usually unknown whether the strains differ at only a single locus (i.e. a comparison between RR vs. SS), at a small number of loci, or whether a significant polygenic component is present. Comparisons made between insects of known genotype are logistically more complex, as they usually require generating a F1 cross, but are much more informative as dominance coefficients can be inferred and both window of selection and window of dominance can be quantified. Our analyses applied to published data indicate highest selection occurs within these windows of dominance (Figures 5,6, S3.5, S3.6).

We have formulated a methodological framework to interpret windows of selection and dominance in diploids. It is important to note that previous authors have identified different parts of this story; for example, that mortality and dominance change over time with insecticide concentration (e.g. Denholm & Rowland, 1992; Gould et al., 2018; Roush, 1989), and that the mortality of different strains or genotypes responds differently over time (e.g. McKenzie & Whitten, 1982; Wanjala et al., 2015) or concentration (e.g. Georghiou & Taylor, 1986; Li, Davey, Miller, Guerrero, & George, 2008). The potential for more persistent insecticides to speed the rate of evolution of resistance was demonstrated more than 30 years ago by computer simulations (Taylor & Georghiou, 1982) and experiments with houseflies (Taylor, Quaglia, & Georghiou, 1983). Our work is the first we know of to attempt quantify changing selection over time or declining insecticide concentration.

### 4.1 Caveats

We have provided a more detailed understanding of the implications of declining insecticide concentrations, but we have still had to make simplifications, and there are, inevitably, caveats to our interpretations. We include little consideration in our analysis of the implications of bioassay reliability, polygenic resistance, competitive release or costs of resistance. We discuss them briefly as follows.

Firstly, mosquitoes do not naturally encounter insecticides in cone and bottle bioassays, so there is some doubt whether findings based on bioassays reflect mortality that occurs in the field (Malima et al., 2008). The use of bioassay data is supported by the fact that the windows of selection shown in Figure 4a are based upon free‐flying mosquitoes in experimental huts (Agossa et al., 2018) and show a very similar pattern to the cone bioassays in Figure 4b. In addition, Churcher, Lissenden, Griffin, Worrall, and Ranson (2016) and Sherrard‐Smith et al. (2018) show evidence that bioassay results are good predictors of mortality in hut trials of insecticidal nets and sprays, respectively.

Secondly, our predictions of selection strength assume resistance is coded by a single gene. We recognize that resistance is frequently a polygenic trait encoded by alleles at many genes, each with a small effect (see discussion in Ffrench‐Constant, Daborn, & Le Goff, 2004; Groeters & Tabashnik, 2000). Theory predicts that polygenic resistance will also generate windows of selection, containing regions of higher selection similar to the window of dominance. We outline this argument further in Appendix S5.

Thirdly, we have only considered the selective advantage of resistance in terms of the reduced mortality of resistant phenotypes. There is also the potential for “competitive release,” where resistant phenotypes have an additional advantage, due to the suppression of susceptibles by a drug or insecticide. Competitive release has been recognized as important for the evolution of drug resistance by parasites within hosts (e.g. Hastings, 2011; Read, Day, & Huijben, 2011), and may also be relevant for insecticide resistance, if competition between individuals is high. Such competition is likely in small breeding pools where larvicides may be applied (Russell et al., 2011), and may potentially drive competition between egg‐laying females for access to quality, sparsely populated breeding sites. Competitive release, where it does occur, could contribute to the length and magnitude of windows of selection by increasing the selective advantage to resistance. We note, however, that the evolution of drug resistance is a different system, where absolute fitness and the ability of a genotype to create enough cells to cause disease and transmission can be more important (Day, Huijben, & Read, 2015) than the relative fitness that is likely to promote the evolution of insecticide resistance.

Fourthly, we have paid little attention to potential fitness costs of resistance, except for noting that our conceptual model of the window of selection can accommodate costs, as a decrease in mortality of resistants below susceptibles to the right of Figure 1a. Fitness costs could similarly be incorporated into plots including heterozygotes, but we chose to exclude them here for simplicity and because the evidence for them is not conclusive (ffrench‐Constant & Bass, 2017). High costs of resistance could create a region at low concentrations, after the window of selection, where resistance is selected against.

Finally, we emphasize that this work focuses only on the evolution of insecticide resistance, which is, of course, not the only measure of the success or failure of an insecticide intervention. If the evolution of resistance was the only concern, then the best strategy would be to use no insecticides at all. Our modelling does not take into account mosquito population change or disease transmission, which would both be expected to increase as insecticide concentrations decline. For a combined modelling approach, including the effect of insecticide interventions on mosquito populations, the evolution of resistance and resulting disease outcomes, see Barbosa, Kay, Chitnis, and Hastings (2018). Note that this earlier work, as with all previous models that we are aware of, did not consider the likely impact of declining insecticide concentrations and the presence of the windows of selection and dominance.

### 4.2 Policy implications and conclusions

We speculate that three aspects of insecticide interventions are most likely to affect the duration, and magnitude, of windows of selection and dominance.

1) Target doses and quality of application, influence whether windows are open on deployment.

2) The rate of decline in concentration, influences when the windows open and close.

3) The interval before the intervention is replaced, influences when windows are closed.

We have demonstrated that selection is highest within the window of dominance. This high selection can be avoided on initial deployment by ensuring that concentrations are high enough to kill all heterozygotes. This is the approach taken in agriculture to maintain the effectiveness of transgenic insecticidal crops (see Appendix S4). Transgenic crops keep producing insecticidal toxins, so declining concentration is not the issue for them that it is for public health. Thus, in public health declining concentration can open the window of dominance, which can remain open until the intervention is replaced.

If interventions are not replaced or reapplied, our work shows that selection for resistance may persist long after deployment, due to continued slight advantages of resistance. Selection can be as intense at low concentrations, when susceptible mortality is low and resistant mortality is zero, as it is at higher concentrations, when susceptible mortality is high and resistant mortality is moderate (Figures 1,5and 6). For example, the Culex data in Figure 6 show an insecticide concentration of 0.0007 ppm gives a higher selective advantage than the concentration 0.06. The corresponding mortalities of RR, SR and SS are 0, 88% and 100% in the first case, and 0, 0 and 24% in the second. Thus, a situation where only 24% of the susceptible mosquitoes are being killed could be selected for resistance more than one where 88% of the SR and 100% of the SS are being killed. This potential of low concentrations to promote resistance has also been demonstrated for antibiotics (Gullberg et al., 2011). Also, note that low insecticide concentrations are likely to lead to more mosquitoes and thus a greater potential for disease transmission and dispersal of resistant mosquitoes.

Bednets too have high potential to promote selection long after deployment, due to their endurance. Nets collected after 7 years use in Tanzania still caused 40%–50% mortality of susceptible strains in a hut trial (Malima et al., 2008) and after 4 years use in Cameroon gave susceptible mortalities of 3%–83% in cone bioassays (Boussougou‐Sambe et al., 2017). While the mortality of resistant strains was not measured, it would be expected to be less than that of susceptible strains, suggesting windows of selection continue to be open after 4–7 years. The current WHO advice (WHO, 2014b) is to keep using bed nets, even if they have already been in use for years, unless a new one becomes available. This is based on the personal protection benefit, from both the physical barrier and some residual (but low) mosquito killing. Currently, WHO documents on measuring and dealing with ageing nets include no consideration of their potential role in selecting for insecticide resistance (WHO, 2011; 2013b; 2014b). Insecticidal effectiveness against susceptible mosquitoes after 3 years is included in initial net acceptance criteria (WHO, 2013a). Mortality beyond this has been removed from considerations of net life because of difficulties measuring it (WHO, 2013c, 2014b).

Our results highlight the importance of considering declining insecticide concentration in the evolution of insecticide resistance. To our knowledge, existing models of the evolution of insecticide resistance have not allowed inputs such as insecticide effectiveness and dominance to change over time (e.g. Barbosa et al., 2018; Birget & Koella, 2015; Levick et al., 2017; South & Hastings, 2018). There are important implications of changing insecticide concentrations for the epidemiology of disease transmission, both directly through altering mortality rates of vectors and indirectly through driving increasing levels of resistance. Recent work has shown both how declining concentrations and levels of resistance influence transmission (Sherrard‐Smith et al., 2018). There remains an important knowledge gap of exactly how declining concentrations are likely to drive the evolution of resistance, threatening the effectiveness of control measures. Different insecticide‐based intervention strategies will each have different sizes of beneficial effects (reducing mosquito populations and disease transmission) and different sizes of detrimental effects (promoting the evolution of resistance). There is a lack of data and understanding to inform such trade‐offs. Management decisions, such as choosing between an intervention with one long‐lasting insecticide or repeatedly applying short‐lasting ones, are not straightforward. In addition, trade‐offs will depend on the timescale of evaluations, that is short‐term impact on disease transmission versus longer‐term impact on resistance and future transmission. These are complex decisions that we do not address here; we simply argue that extensive windows of selection and windows of dominance will, almost inevitably, arise in public health deployments of long‐acting insecticides and that these windows will need to be incorporated into such evaluations. We agree with Huijben and Paaijmans (2017) that a greater understanding of the evolutionary processes causing resistance is needed to develop better strategies to manage it. We have shown how the forces driving the evolution of resistance can be usefully documented, interpreted and quantified in terms of windows of selection and dominance. We argue that focusing attention onto the relative mortalities of resistant, susceptible and heterozygous genotypes, over time, is necessary to inform strategies to reduce the evolution of resistance to existing and new insecticides.

## CONFLICT OF INTEREST

The authors declare no competing interests.

## Data Availability

The data and R code to produce all of the figures in this paper are available at http://doi.org/10.5281/zenodo.3533012 with supporting code at http://doi.org/10.5281/zenodo.3533001 and http://doi.org/10.5281/zenodo.3533010.
