# Supplementary material for: The role of windows of selection and windows of dominance in the evolution of insecticide resistance in human disease vectors
Source: Evol Appl. 2019 Dec 10;13(4):738–51. doi: 10.1111/eva.12897 (PMC7086049; doi:10.1111/eva.12897)

**Supplementary information**

The role of windows of selection and windows of dominance in the evolution of insecticide resistance in human disease vectors

Appendix S1. Methods for experiments carried out in Liverpool

Appendix S2. Additional results and plots

Appendix S3. Additional windows of selection replotted from the literature

Appendix S4. Resistance management lessons from transgenic (Bt) crops

Appendix S5. How resistance encoded by single genes or by polygenes affect the impact of windows of selection

**Appendix S1. Methods for the experiments carried out in Liverpool**

*1.1 Deltamethrin spray: Windows of selection in units of concentration*

Whatman filter paper No. 1 was cut into 12cm x 15cm pieces, which were impregnated with deltamethrin by making up a solution of the required concentration in acetone and silicon oil and applying 1.98ml to the surface of the filter paper using a pipette to dispense drops evenly across the whole paper. Papers were then hung up in a fume hood to dry overnight before use in bioassays. Two ranges of deltamethrin concentrations were produced for use in cone bioassays, firstly 0.00125%, 0.0025%, 0.005%, 0.01%, 0.02%, 0.04% and 0.08%, and then to a concentration range 10 times higher, namely 0.0125%, 0.025%, 0.05%, 0.1%, 0.2%, 0.4% and 0.8%.

Insecticide susceptible Kisumu and resistant Tiassalé strains of *Anopheles gambiae* were exposed to each deltamethrin concentration using the WHO Cone bioassay (WHO/CDS/NTD/WHOPES/GCDPP/2006.3). Ten, 2-5 day old, mated but not blood fed, female mosquitoes were aspirated into a plastic cone plugged with cotton wool, applied to each paper, held on a board at 45 degrees, and left for 30 minutes before being aspirated off and held in a stability cabinet at 27°C ± 2 °C and 70% ± 10% relative humidity for 24 hours, with access to a 10% sugar solution provided on cotton wool. Mosquitoes were scored for knockdown (the proportion of incapacitated mosquitoes at the end of the 30 minute exposure) and mortality 24 hours (± 2 hours) after exposure. Five replicate papers of each concentration were treated and used for bioassays, alongside five replicate negative control papers, treated with acetone and silicon oil only.

*1.2 Deltamethrin spray: Windows of selection in units of time*

Three types of surfaces were treated, representative of materials which may be used to construct dwellings in areas of IRS application: 12×12cm squares of untreated beech, blocks of cement, at least 5 mm thick, prepared in 10 cm Petri dishes and allowed to dry for at least 30 days prior to testing commencing, and 10cm diameter mud tiles. A mud house brick, obtained from partners at the Institut de Recherche en Sciences de la Santé (IRSS), Burkina Faso, from their field site in Vallée du Kou 7 (VK7): 4°24′W and 11°24′N, was broken down into dust, reconstituted using purified water to fill a mould, and stored in a climate-controlled stability cabinet (27°C ± 2 °C and 80% ± 10% RH) for a minimum of 30 days to ensure the moisture level drops to 4 – 5%, to produce a smooth mud surface.

K-othrine Deltamethrin WDG (WHO approved grade) was applied to each surface type, using a ‘Potter Tower’ (Potter Precision Laboratory Spray Tower, Burkard Scientific), at a rate of 25 mg AI/m^2^ (diluted in deionised water), alongside negative control surfaces of each type which were sprayed with deionised water only. The Potter Tower is internationally recognised as the standard reference for chemical spraying techniques in the laboratory. The spray apparatus is designed to give an even, predictable deposit over a circular area up to 11cm in diameter. Prior to use the Potter Tower is calibrated, to ensure less than 10% variation in spray density across the treated surface, and less than 10% variation in spray weight between applications.

Bioassays were performed with insecticide susceptible and resistant strains, respectively, of three vectors of human disease held by LITE, Liverpool School of Tropical Medicine: *Anopheles funestus* (Fang and Fumoz), *An. gambiae* (Kisumu and VK72014), and *Ae. aegypti* (New Orleans and Cayman). At each time point, 10, 2-5 day old, mated but unfed, female mosquitoes were exposed to each surface using the WHO Cone bioassay (WHO/CDS/NTD/WHOPES/GCDPP/2006.3). Mosquitoes were aspirated into a plastic cone plugged with cotton wool, applied to each surface, held on a board at 45 degrees, and left for 30 minutes before being aspirated off and held in a stability cabinet at 27°C ± 2 °C and 70% ± 10% RH for 7 days, with access to a 10% sugar solution provided on cotton wool. Mosquitoes were scored for knockdown (the proportion of incapacitated mosquitoes at the end of the 30 minute exposure) and mortality, daily, for 7 days after exposure. Bioassays were repeated at 24 hours and 1, 3, 5, 7, 9, 12 and 18 months after treatment of the surfaces, or until mean mosquito mortality 7 days after exposure dropped to below 60% on two successive months. The same surfaces were used for bioassays at each time point, but both strains of each species were exposed to a different set of treated surfaces, 3 replicates per surface type, treated with Deltamethrin and equivalent negative controls. After spraying, and between bioassays, the treated surfaces were stored in a climate-controlled stability cabinet (27°C ± 2 °C and 80% ± 10% RH), vertically and unsealed, with air circulation and in the dark.

**Appendix S2. Additional results and plots.**

In this section, we provide additional plots to substantiate results and discussion surrounding our theoretical analyses of windows of selection, and our laboratory experiments.

Figure S2.1 shows the effect, of changing insecticide exposure, on selective advantage and simulation time to resistance shown in Figure 1 C & D. Figure S2.1 uses exposure levels (proportion of all mosquitoes exposed to the insecticide) of 0.5 and 0.1 where Figure 1 uses 0.3. Given that males do not feed on blood, and are less likely to encounter insecticides in bednets or sprayed on house walls, then a value of 0.5 is likely to be close to the upper limit experienced in the field. Comparing Panels B and D shows that selective advantage of resistance is lower when exposure is lower, but that the qualitatitve pattern of selective advantage, being higher within the window of dominance, remains the same. Similarly comparing panels C and D, times to resistance are higher at lower exposures, but the qualitative pattern, of resistance evolving fastest within the window of dominance, is consistent.

Our plots (e.g. Fig 1C) show selective advantage of resistance (z) at any given point in the Window of selection, in isolation to other points. For example, in Figure 5 we calculate z at 1% permethrin concentration assuming a proportion *x* of the population (30% in our example) is encountering 1% permethrin and the remaining 70% encounters none. Similarly, when calculating z at 0.1% we assume 30% of the population encounter 0.1% permethrin and 70% no insecticide. This enables us to quantify how selection changes within the window of selection, by directly comparing each point under the same exposure levels. In reality, the 30% of the population exposed would be encountering a range of different concentrations. This raises the possibility that the area under the curve (AUC) of the selection/time plot may allow a quantitative metric of overall selection for resistance. Such a metric could allow a quantitative comparison of how different insecticide deployments are expected to drive resistance. We could imagine quantifying the window of selection of an intervention by measuring how much selection for the resistance allele is applied, compounded over insect generations, until the insecticide becomes ineffective. However, care should be taken. This metric will be affected by initial starting frequency if resistance is recessive, because it determines the proportion of R alleles in the RR homozygotes. Starting frequency will also influence the time taken for frequency to reach 100% when no further selection can take place even if the window is still open. This AUC approach, therefore, appears a plausible metric to quantify how windows of selection drive resistance, but requires substantial further investigation and validation that lie outside the scope of the present manuscript.

Figures S2.2-S2.3 show additional results from the experiment illustrated in Figure 3 of the main manuscript. This experiment investigated mosquito mortality in the months after spraying deltamethrin onto three surface materials. It did this for resistant and susceptible strains of three mosquito species. Figure 3 of the main manuscript showed the mortality of mosquitoes, 24 hours after they were exposed to the insecticide treated surfaces. Figure 3 showed windows of selection that were open for the length of the study period (18 months) and limited evidence for susceptible mortality starting to decline on some species/surface combinations by 18 months.

Figure S2.2 shows an equivalent plot to Figure 3, but displaying knockdown at 30 minutes, instead of 24 hour mortality. 30 minute knockdown declines sooner than 24 hour mortality. Many of the mosquitoes, that aren't knocked down by 30 minutes, go on to die before 24 hours. This shows that it can take the insecticide longer to act when it has been sprayed on a surface months previously, than when it is freshThis is one indication of the declining insecticide concentration. For the window of selection, the 24 hour mortality is more relevant because, although susceptible mosquitoes in later months on mud and cement are starting to survive longer than 30 minutes, they are still dead by 24 hours and under a selective disadvantage to the resistant strains.

Figure S2.3 shows the mortality of the control mosquitoes that were not exposed to the insecticide. The mortality of resistant and susceptible strains not exposed to the insecticide did not differ consistently in the months of the experiment or change over time. This confirms that the insecticide was responsible for the differences between susceptible and resistant mortality, and that the insecticide generated the windows of selection.

**Figure S2.1.** Changes to selective advantage and time to resistance, through the window of dominance, under different exposure levels. Equivalent of Figure 1 in the main manuscript which uses an exposure level of 0.3, panels B & C use an exposure of 0.5 (half of all mosquitoes exposed to the insecticide), panels D & E use an exposure of 0.1. The x-axis is shared between panels A-F. Panel A illustrates how the mortality probabilities change for each genotype and the row of numbers along the top of the plot is the dominance of resistance at each time point. Panels B & D show how selective advantage over one generation changes during the windows of selection in panel A (see methods for calculation). Panels C & E show predicted time until resistance allele frequency reaches 50% for simulations started at each point in panel A.

**Figure S2.1 (legend above)
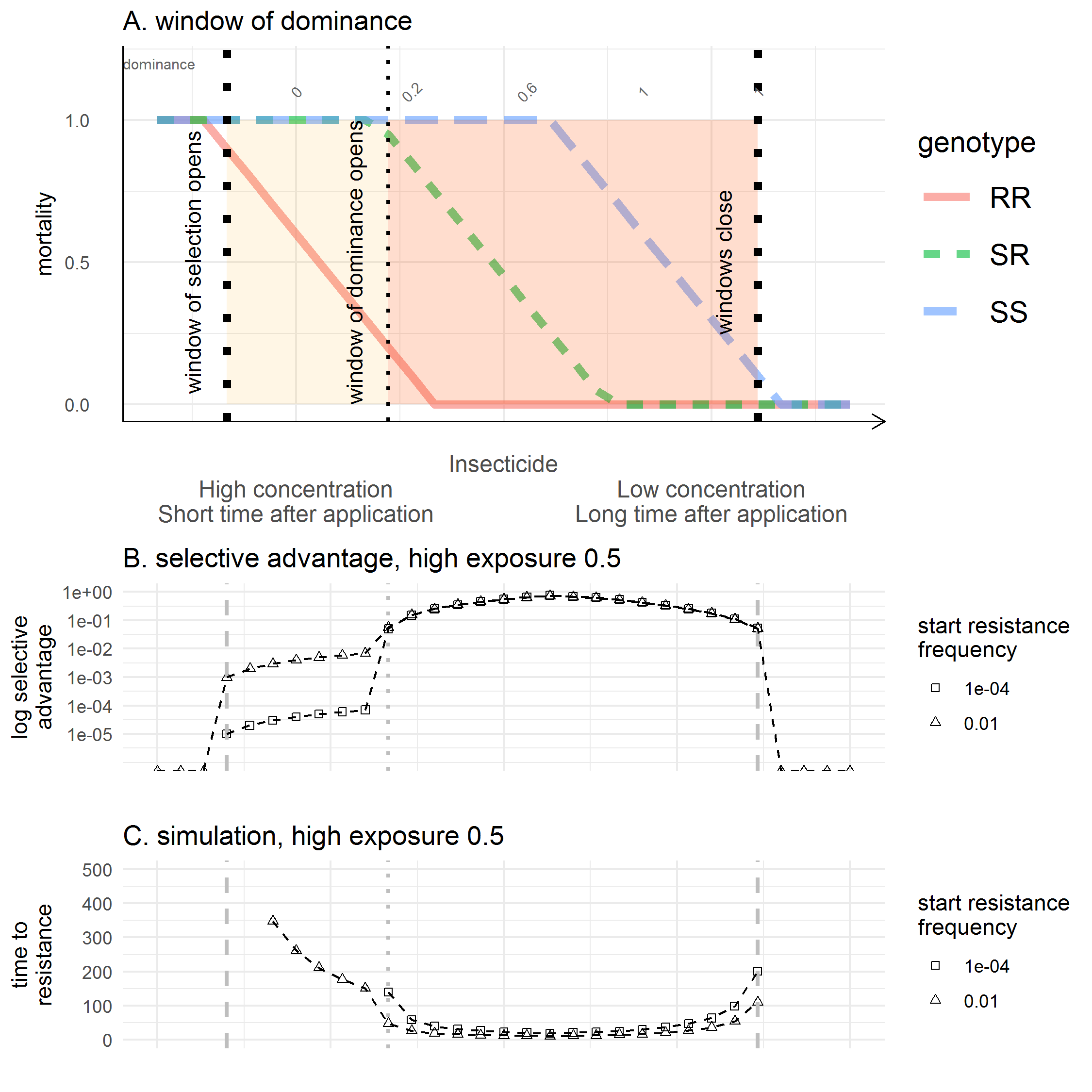

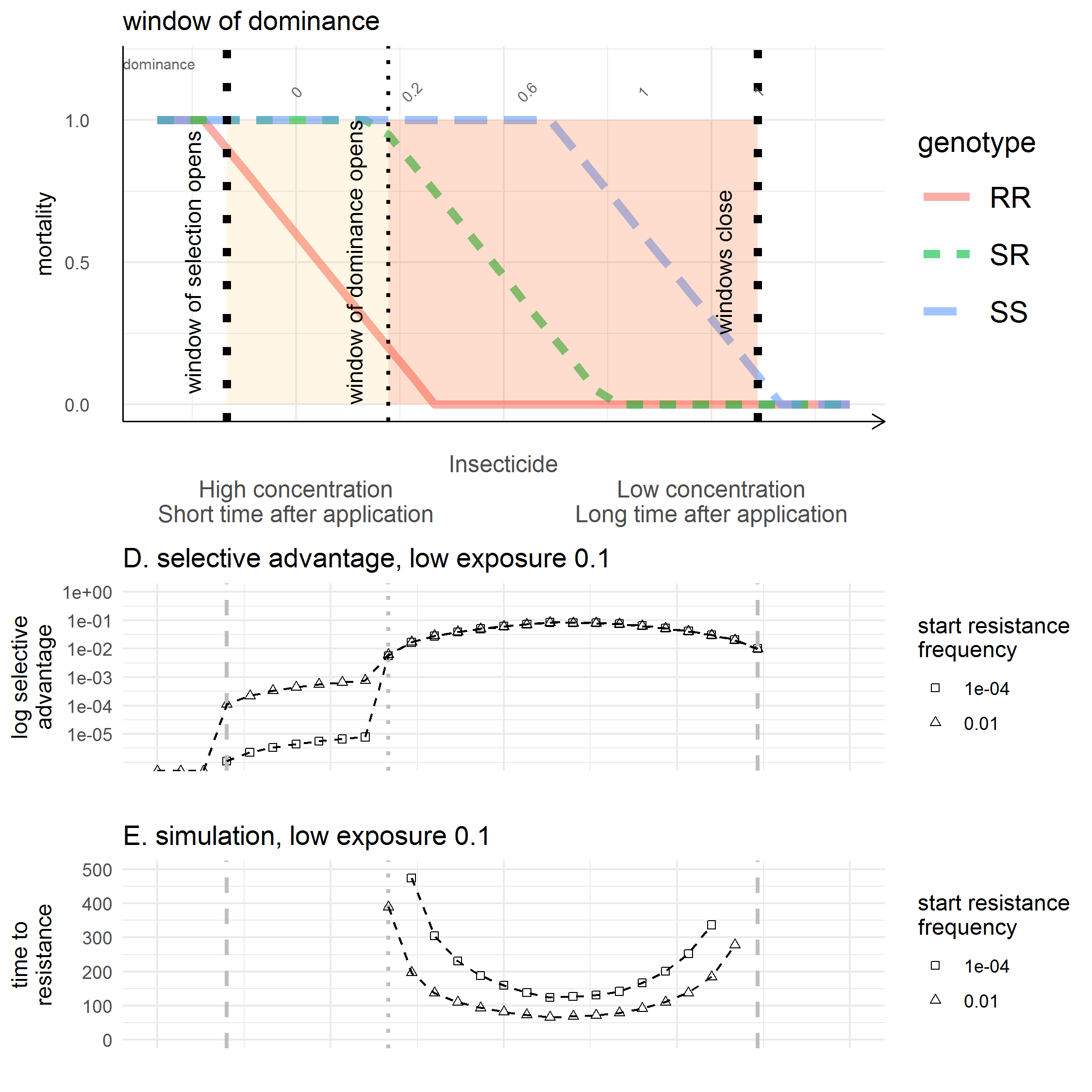
**

**Figure S2.2.** Thirty minute knockdown in the months after deltamethrin spraying for three vector species; *Anopheles gambiae*, *Anopeheles funestus* and *Aedes aegypti*. Equivalent of Figure 3 in the main manuscript, which shows 24 hour mortality. Points show the percentage knockdown of 3 replicates for resistant and susceptible strains, and lines show a locally weighted smoother (loess). Deltamethrin was applied to three different substrates (cement, mud, and wood tiles), which were then kept in an incubator to mimic African field conditions between assays. Knockdown was measured 30 minutes post-exposure in cone bioassays applied to the tiles.


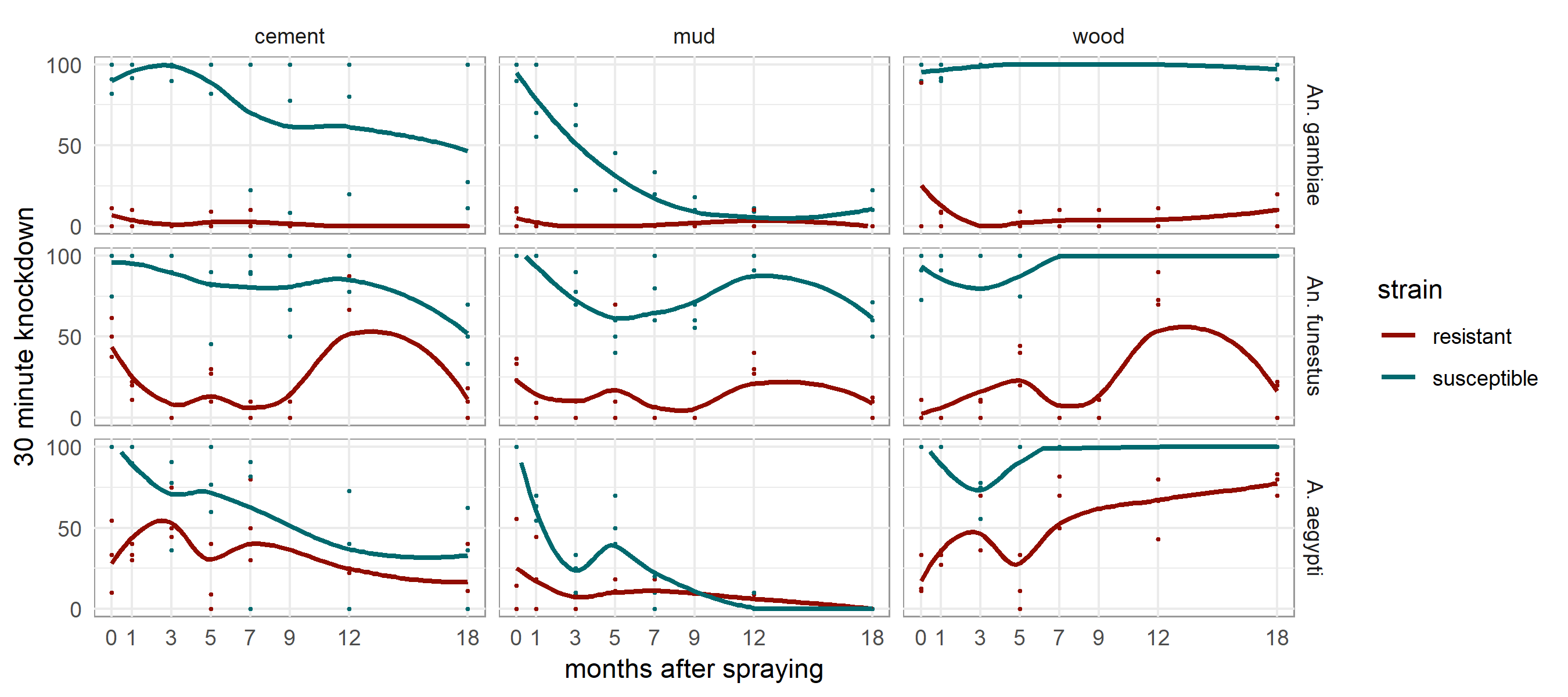


**Figure S2.3.** Control mortality in the months after the experiment start, for three vector species; *Anopheles gambiae*, *Anopheles funestus* and *Aedes aegypti*. Equivalent of Figure 3 in the main manuscript, which shows 24 hour mortality on tiles sprayed with deltamethrin, except that these tiles were unsprayed. Points show the percentage mortality of 3 replicates for resistant and susceptible strains and lines show a locally weighted smoother (loess). The tiles were kept in a stability chamber set to mimic African field conditions between assays. Mortality was measured 24 hours post-exposure in cone bioassays applied to the tiles.


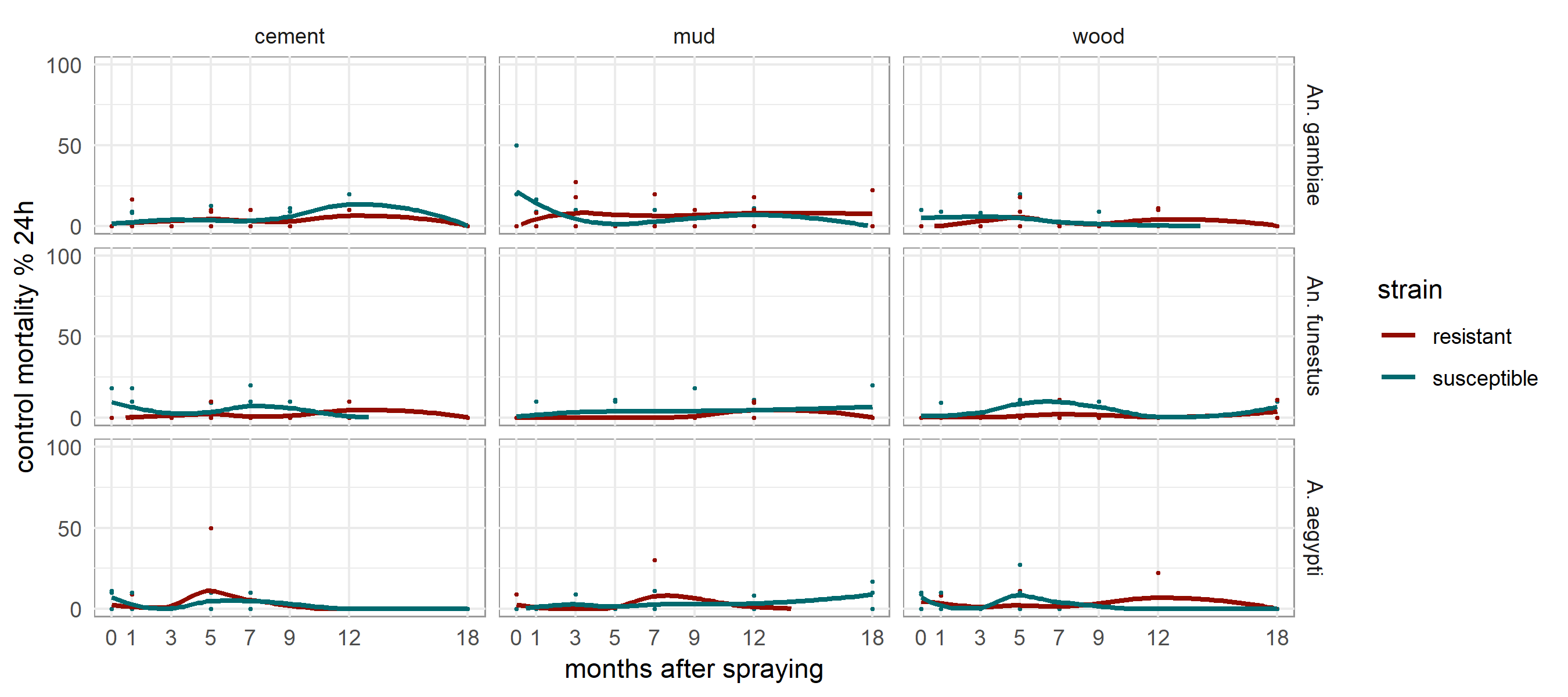


**Appendix S3. Additional windows of selection and dominance extracted from the literature.**

We searched Medline, Web of Science, and Global Health databases with no date limitations. The following two search terms were used, noting that the term insecticid* will recover “insecticide”, “insecticides”, and “insecticidal” and the term resistan* will recover both “resistance” and “resistant”.

Search (1):

“Window of selection” AND insecticid*

Returned no hits

Search (2) ;

Insecticid* AND persistence AND resistan* AND (malaria OR Dengue OR “vector borne” OR Anopheles OR Aedes)

The terms in parenthesis are required to exclude the large number of agricultural publications. This search returned 359 hits.

The first search was designed to confirm that the evolution of insecticide resistance has not previously been explicitly discussed in terms of windows of selection. The second search was designed to confirm that an appreciation that long persistence of insecticides drives resistance has been in the literature for a long time (e.g. Taylor & Georghiou, 1982). We then scanned this literature for studies that quantified a window of selection rather than speculating on its existence i.e. those reporting insecticide mortalities of resistant and susceptible genotypes over ranges of concentration and/or time and, in particular, examples for single-locus mutations so we could examine not just windows of selection, but how dominance and selection for resistance changed within them. We then read the citations in the REX consortium publications (REX Consortium, 2013; REX Consortium, 2016; this consortium was explicitly assembled to investigate how different fields of resistance research interact). The examples that we plot in the main manuscript and in this SI were read in detail and we read both the papers they cite, and papers which subsequently cited these studies. Finally, we informally asked colleagues with specialist knowledge of insecticide resistance if they could suggest publications matching our needs.

The papers found with data allowing calculation of windows of selection are summarised in Table 1 of the main text, and here we provide a description of these experiments and plots.

Agossa et al. (2018) investigated time after spraying rather than concentration and reported mortalities of both free flying mosquitoes in hut trials and individuals placed in cone bioassays against walls sprayed with either deltamethrin or clothianidin (Sumishield). They did this for a susceptible strain and the local strain. Resistance to deltamethrin in the local strain was indicated by <30% mortality to deltamethrin in a bioassay at the standard concentration and a frequency of resistance alleles of 95% (30 individuals tested for kdr gave 27 RR, 3 SR and no SS). Their data are replotted to show the windows of selection in Figure 4 of the main manuscript. The windows of selection for deltamethrin are shorter than in the equivalent laboratory experiment summarised in Figure 3. This may be due to different strains being used in the assay or that the hut walls were subject to more realistic processes, such as people brushing against them, than the idealised environment of a laboratory incubator. Free flying mosquitoes in hut trials (Fig 4A) suggested a window of selection of at least 8 months and those in the cone bioassays (Fig 4B) suggested 5 months. The exact nature of the window of selection for deltamethrin differs only slightly according to whether it is assessed by mortality at 24 hours or 5 days. Using 5 day mortality is advisable for clothianidin given its slow mode of action (Agossa et al., 2018). Resistance is not suspected a priori for this newer insecticide and its window of selection does seem to be closed at the start as expected. However worryingly a window of selection for clothianidin resistance does appear to be opening by month 6 to 8 post-application (Fig 4).

Anshebo et al. (2014) demonstrate that windows of selection can occur in insecticidal nets as well as for sprays. They report the mortality of *Anopheles* mosquitoes exposed to insecticidal nets distributed in Ethiopia; their data are replotted in Figure S43.1. This study is particularly useful because they both investigated mortality over time after net distribution and measured the insecticide concentration within nets. Insecticide concentrations declined by around 25% in the first year and then levelled off, only declining by around a further 5% in the next year and half. Interestingly there was a large variation in the concentrations measured from nets across all time periods. At 3-6 months susceptible mosquitoes showed near 100% mortality across all concentrations and two of the four locally-caught samples had mortality less than 75%. At 14-20 months locally-caught mosquitos showed lower mortality than the susceptible strain and that difference was greater at medium to low insecticide concentrations. At low concentrations the mortality of the susceptible strain also declined from 100%. At 26-32 months there was a greater difference between the susceptible and local strains across all concentrations resulting from a greater decline in the locally caught mosquitoes. The authors suggest this decline is evidence for a rapid increase in resistance in the area. It could also indicate a decline in the efficacy of the nets over time that is not captured by the concentration measurements. Either way there is evidence for windows of selection being open at 6 months, further open at 14 months and continuing to be open at 32 months when the study ended.

Bagi et al. (2015) compared the mortality of three resistant strains to one susceptible one across a range of permethrin concentrations (Fig S3.2). Their results were consistent with our results presented in Figure 2 i.e. there is a wide range of concentrations for which the susceptible mortality is much greater than the resistant. There is relatively little difference between the three resistant strains. The most resistant lab strain Tiassalé exhibited the lowest mortalities at intermediate concentrations suggesting the widest window of selection.

Etang et al. (2016) showed that insecticide concentrations in nets can decline through washing and this could alter the window of selection. Resistant and susceptible mosquito strains were exposed to net samples washed a different number of times and mortality differences measured. Mortalities were compared to standard bottle concentration bioassays also on the resistant and susceptible strains. Figure S3.3A shows the replotted concentration bioassays and Figure S3.3B the mortality after 24 hours according to the number of washes of the net samples. Both plots show a window of selection that is initially closed at the highest concentration or lowest number of washes and opens with declining concentration or increasing number of washes. In both cases the window remains open at the lowest concentration or highest number of washes, with susceptible mortality still at 100%, suggesting that the window would remain open for longer. If 1 hour knockdown (the proportion of incapacitated mosquitoes after an hour) is used instead of 24 hour mortality as an estimate of mortality in the field, the window is much smaller and doesn't open until later (Fig S3.3C). This could be the case if the knocked down mosquitoes were all killed by arthropod predators under field conditions.

Mahama et al. (2007) investigated the effect on net mortalities of time hanging in local houses in Côte d’Ivoire. They exposed susceptible and resistant lab strains to net samples in cone bioassays. The entire time series is not provided in the paper but the textual description allows the state of the window of selection at the start and end of the study to be inferred. Susceptible strain mortality was consistent at 99-100%, where the resistant strain mortality was 69-75% in the first 3 months declining to 2% by month 15. The difference in mortality in the first 3 months suggests that the window of selection was already open when the nets were distributed. The 97% difference between susceptible and resistant mortality at month 15 suggests the window of selection is maximally open at this stage and likely to stay open for many more months.

The first two studies presenting data on all three genotypes (SS,SR &RR), thus enabling quantification of selection, are described in the main paper (Li et al., 2008, Georghiou & Taylor, 1986). The other two are described briefly here. Corbel et al. (2004), working on Anopheles across a smaller insecticide concentration range than the first two, showed that the mortalities of all 3 genotypes declined together and the window of dominance is open for all concentrations except the lowest (Fig S3.5)

McKenzie & Whitten (1982) investigated the sheep blowfly and quantified the duration of protection afforded by insecticides externally applied to sheep. They presented relative rather than absolute mortalities so the data cannot be used to estimate selective advantage or time to resistance, nevertheless they do indicate windows of dominance with interesting differences (Fig S3.6). For dieldrin (left panel of Fig S3.6) dominance values are higher as the SR mortality is closer to the RR. For diazinon dominance is lower as the SR mortality stays closer to the SS. This lower dominance is compatible with the observation that resistance to diazinon developed more slowly than dieldrin in the field (McKenzie & Whitten, 1982).

**Figure S3.1.** Window of selection in time and concentration for *Anopheles* mosquitoes on insecticide treated nets deployed in Ethiopia and collected over three time intervals. Replotted from Anshebo et al. (2014), their Figure 6 with additional data for wild mosquitoes added from the authors for months 3-6. Concentration was estimated by X-ray fluorescence and mortality was assessed using net samples in CDC modified WHO cone bioassays. The three panels correspond to these three time intervals i.e. 3-6, 14-20 and 26-32 months. The susceptible strain was mostly *Anopheles gambiae* Kisumu in months 3-6 and *Anopheles arabiensis* Nazareth in the other time periods. The wild strain came from local caught *Anopheles* larvae. The four wild data points for months 3-6 were not included in the original paper due to small sample size they are included here as they give some suggestion of a difference between susceptible and wild mortality even in the first time period. Concentration is plotted on a declining scale for consistency with other plots. Concentrations decline from the first time interval (i.e. 3-6 months post deployment) and then level off to reach about the same values in both the 14-20 month and 26-32 periods. The final interval shows large differences between the mortality of wild caught and susceptible mosquitoes that are greater at lower concentrations.


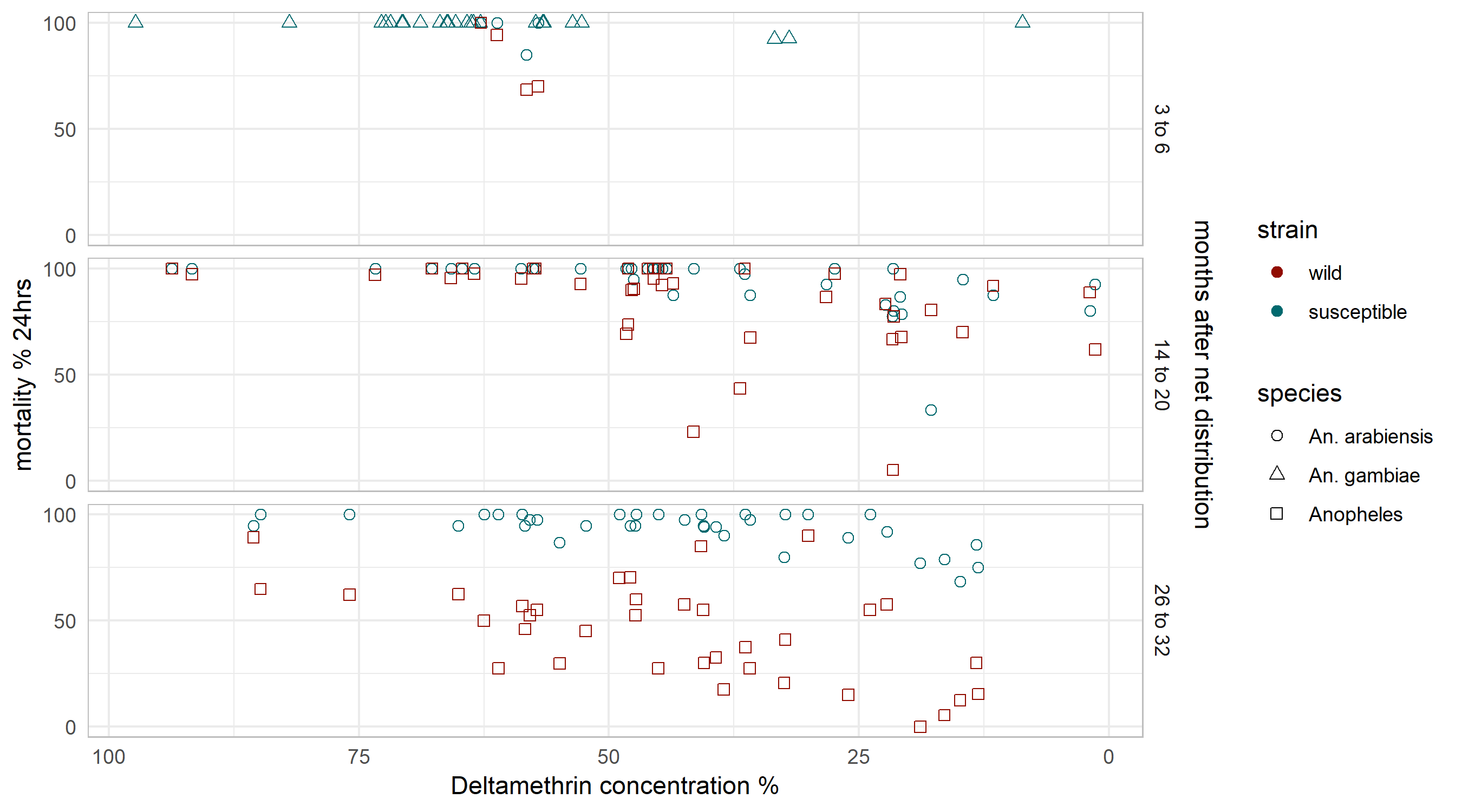


**Figure S3.2.** Windows of selection in units of concentration for different strains of *Anopheles gambiae* s.l. exposed to permethrin. Mortality was measured at 24 hours after exposure to different concentrations of permethrin in CDC bottle assays. Data replotted from (Bagi et al., 2015). The x axis is plotted from high to low concentrations for consistency with time plots given that concentrations decline with time after deployment. Points show the percentage mortality of 4 replicates for resistant and susceptible strains and lines show a locally weighted smoother (loess). The results suggest that windows of selection are open except at the highest concentrations tested.


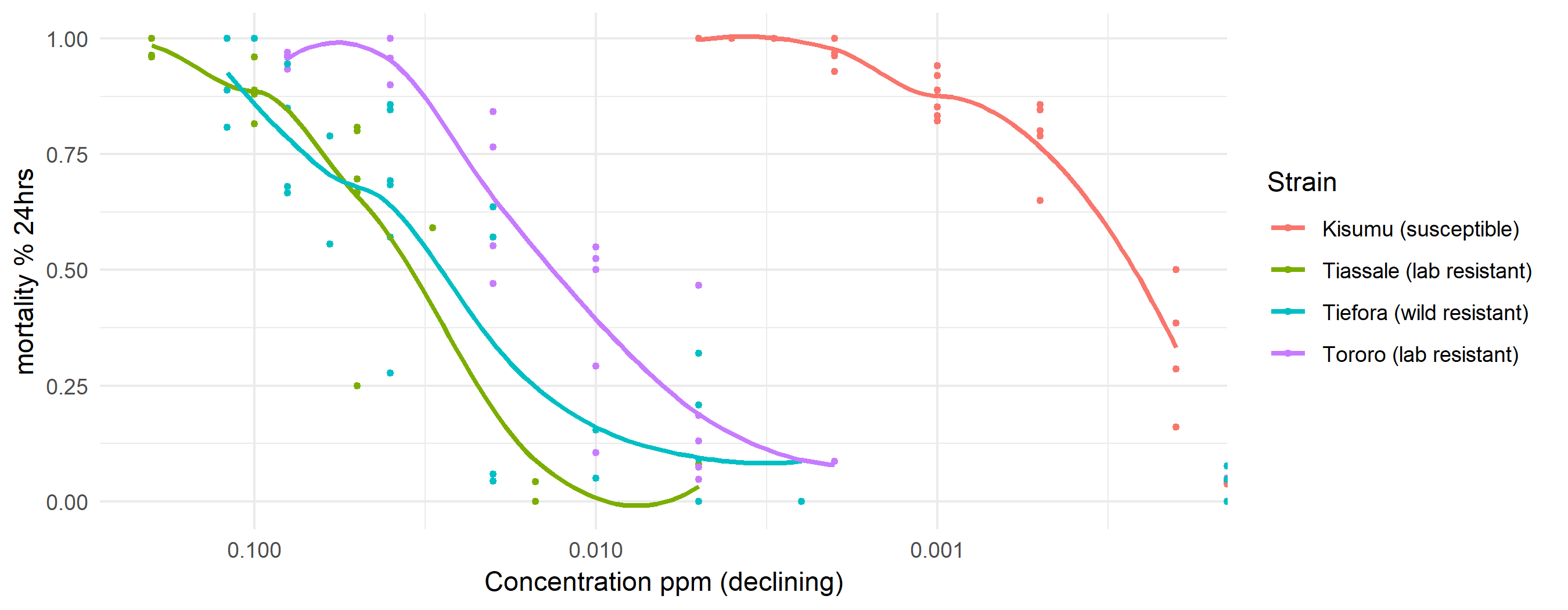


**Figure S3.3.** Windows of selection in units of concentration for *Anopheles* mosquitoes exposed to deltamethrin and in units of washes for mosquitoes exposed to bed-net samples. Mortality of deltamethrin susceptible *Anopheles gambiae* and the local strain in Cameroon; data from Etang et al. (2016). Concentration tests were done using WHO tube assays, the effect of washing was estimated using WHO cone bioassays on net samples provided by the manufacturer. A. The windows of selection as a function of concentration. The Window opens once concentration falls below 0.1% and the lowest concentrations investigated were still associated with differential mortality suggesting that the window remained open for at least a 100-fold range of concentrations. B. Impact of net washing on 24 hour mortality. This metric suggests that net washing similarly causes the window of selection to open by reducing the mortality of the local strain but not the susceptible one. C. The impact of net washing on 1 hour knockdown, which declined less than 24hour mortality.

**Figure S3.3. (legend above)**


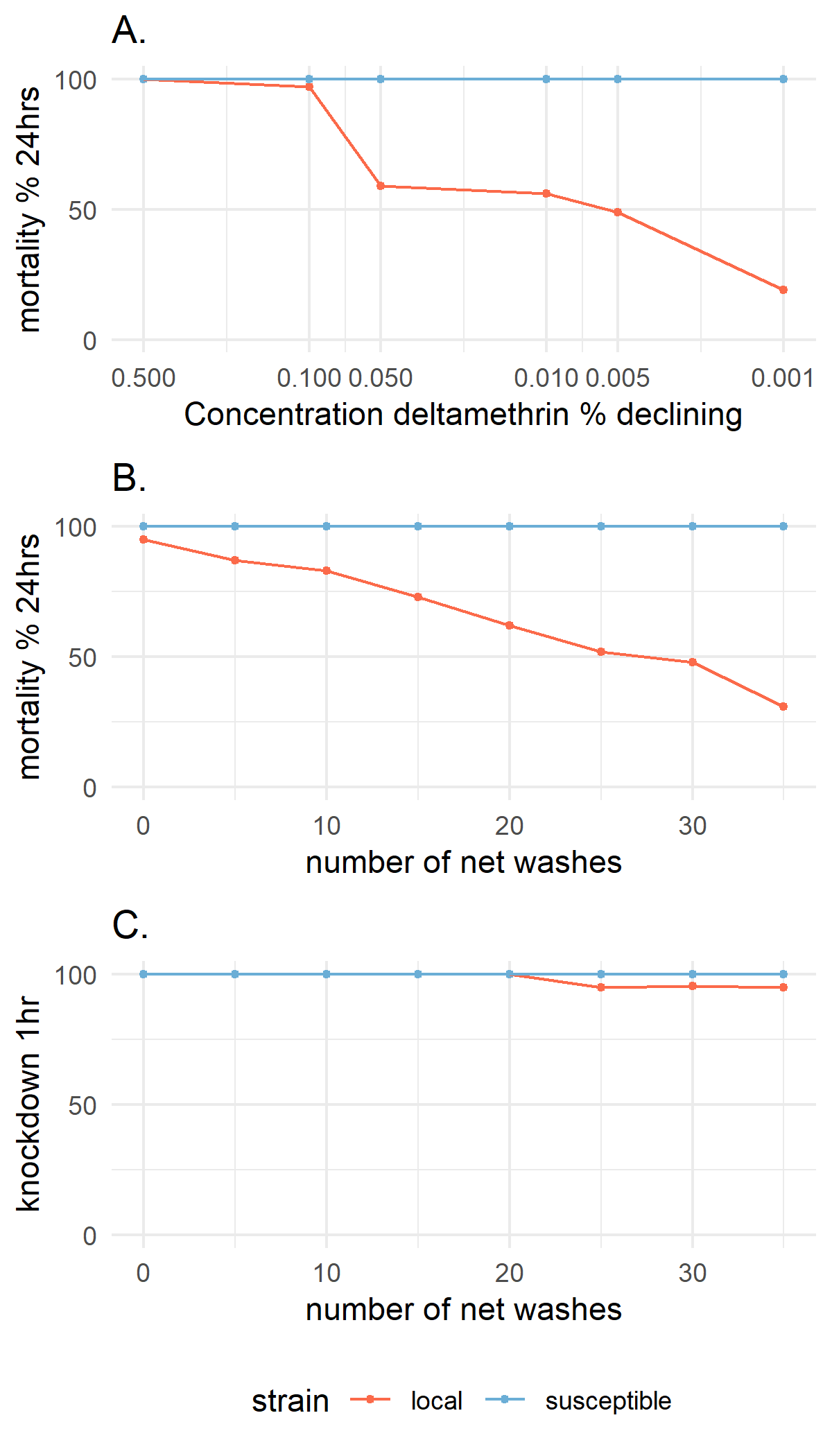


**Figure S3.4**. Window of selection in units of time for *Anopheles gambiae* exposed to deltamethrin treated nets three and 15 months after distribution. Results are from cone bioassays (mortality after 24 hours) conducted on susceptible and resistant lab strains exposed to fabric samples from nets distributed in Côte d’Ivoire. Data replotted from description in the text of Mahama et al. (2007).


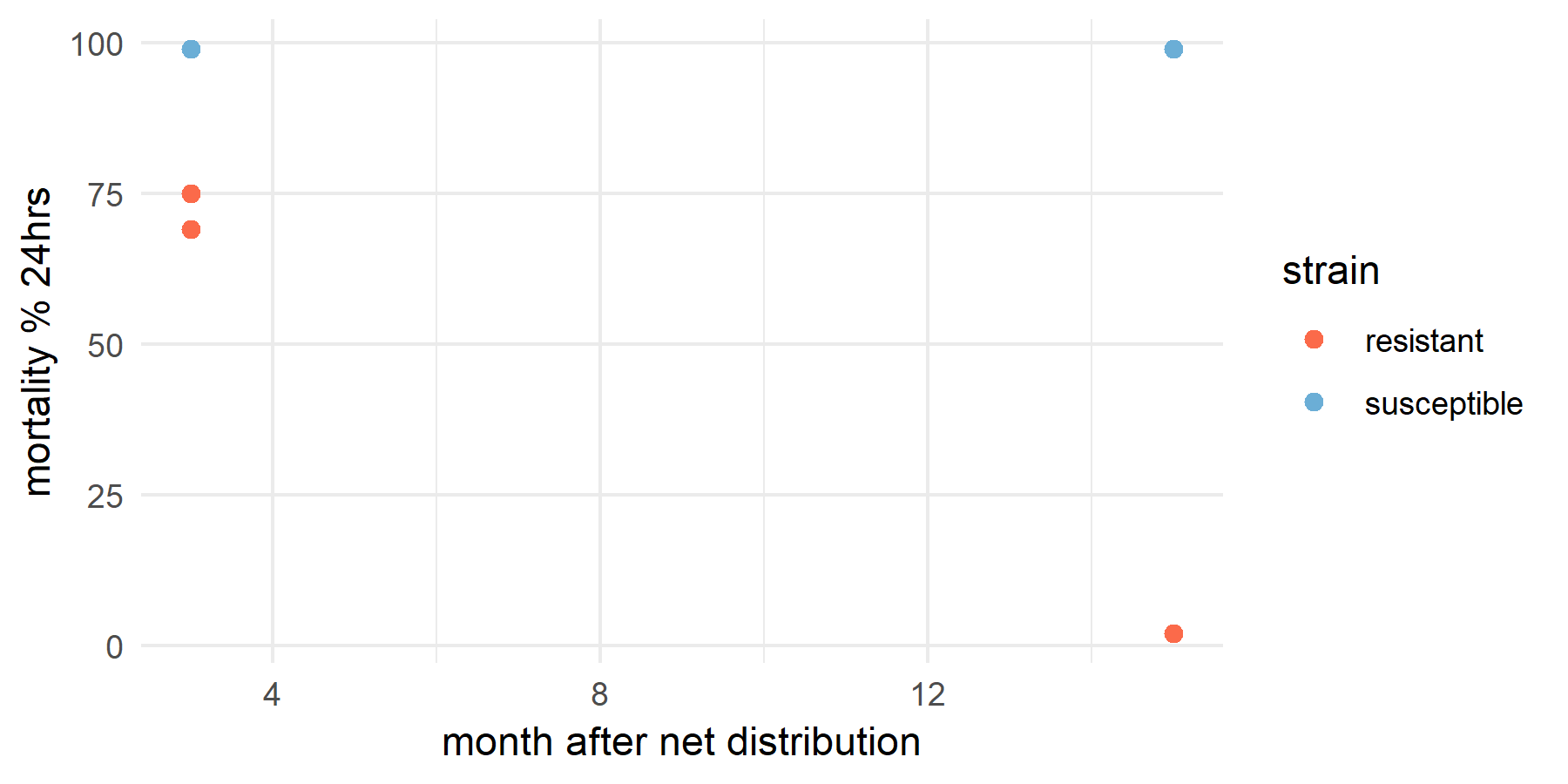


**Figure S3.5.** Windows of selection and dominance in units of concentration associated with permethrin resistance in *Anopheles gambiae* exposed to treated nets. Panel A shows the mortality of mosquitoes exposed to treated netting in tunnel tests; points show the mean of three replicates from data reported in Figure 2 of Corbel et al. (2004). The row of numbers along the top of panel A is our calculation of dominance of resistance for each concentration. Panels B and C show our measures of selection calculated on the same concentration x axis. Panel B show how selective advantage within a single generation changes during the windows of selection and how it depends on the starting frequency of resistance. Panel C shows simulation results of the number of generations needed to reach a resistance allele frequency of 50%.
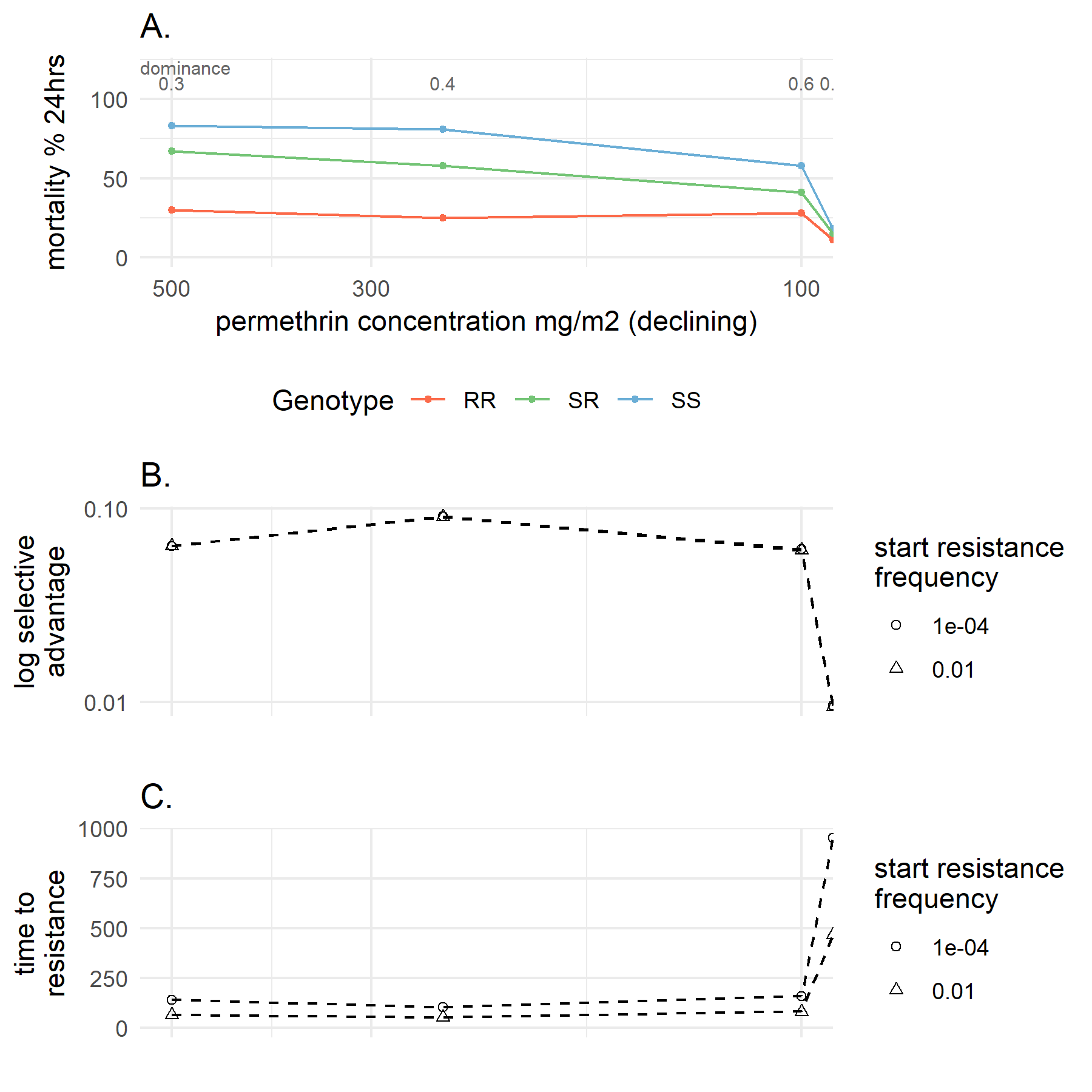


**Figure S3.6.** Windows of selection and dominance in units of time obtained from sheep sprayed with either Dieldrin or Diazinon and experimentally inoculated with eggs of the Australian sheep blowfly, *Lucilia cuprina*; the eggs were an F2 cross from homozygous grandparents to give all 3 genotypes. Surviving larvae were collected after 48 hours and exposed to diagnostic concentrations of insecticide to establish whether they were RR,SR or SS. This was repeated on control sheep that hadn't been treated and the proportional difference between the frequency of genotypes on the treated sheep and controls was calculated. The relative viability of each genotype was obtained by dividing this proportional difference by the maximum in the timestep. These plots show how relative viabilities change over time and can be used to calculate changing dominance. Note that the genotype viabilities are relative, not absolute, so cannot be used to estimate selection in the same way that we did in the previous two examples. Data replotted from McKenzie & Whitten (1982) and the row of numbers at the top of the plots are the effective dominance of resistance at each time point.


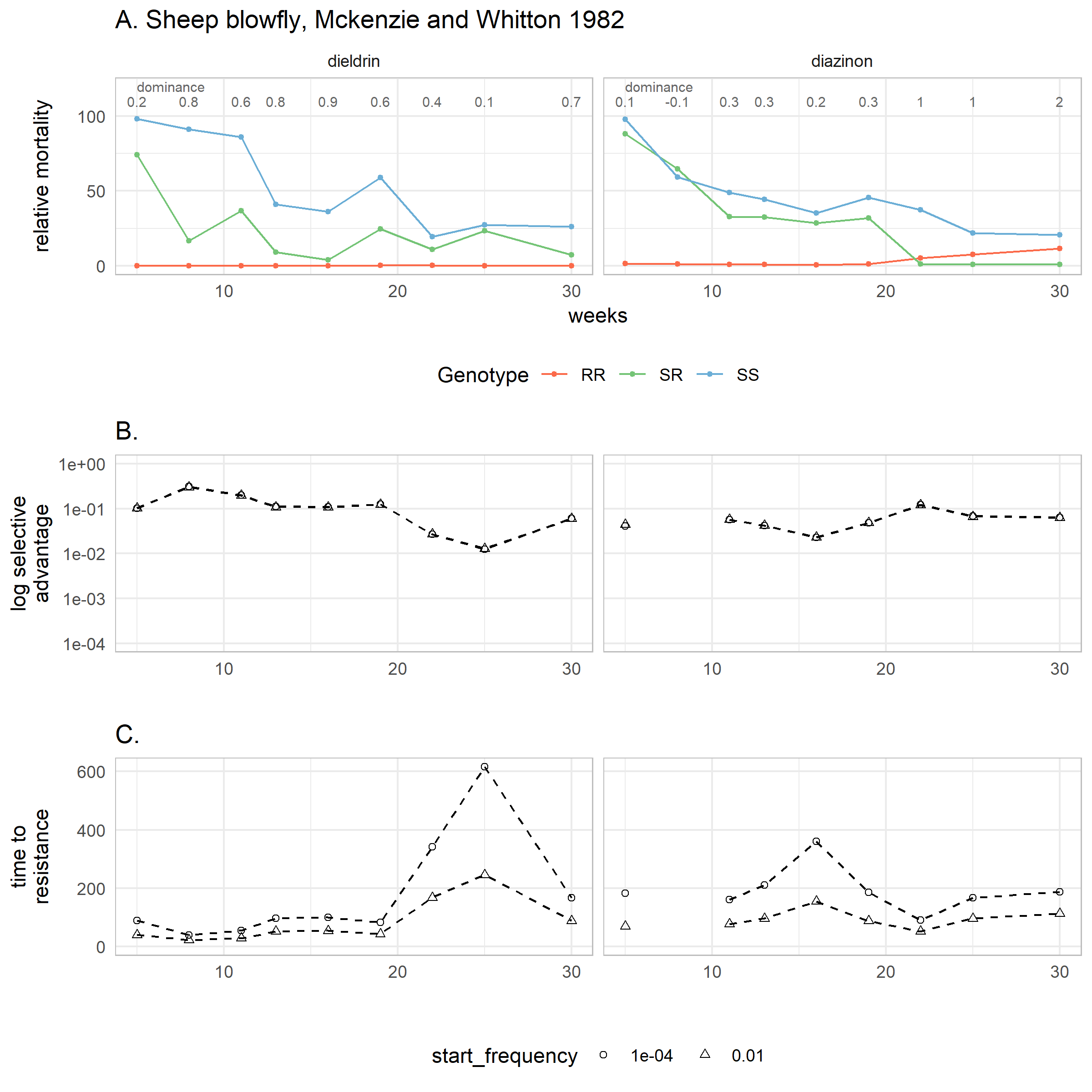


**Appendix S4. Resistance management lessons from transgenic Bacterial toxin (Bt) crops**

Bt crops provide useful lessons for our discussion of declining insecticide concentrations. These plants are genetically modified to produce insecticidal toxin(s) as they grow, so, unlike externally deployed insecticides, concentrations do not decline. This provides the genetic “designers” the opportunity to control expression levels of toxins, with the deliberate intention of minimising selection for resistance (Tabashnik & Carrière, 2017). The most obvious “design” question is what concentration should be expressed and this evoked debates and recommendations that implicitly recognised the need to avoid windows of selection and dominance.

Bt crops have been hugely successful over the past 20 years and planted in hundreds of millions of acres (Tabashnik & Carrière, 2017). The commonest strategy is called the 'high-dose refuge strategy'. This requires high levels of toxin expression, so that a high dose is presented to insects feeding on the plants (which is accompanied by refuges in which pests are not exposed to the toxins). There is a 'high-dose standard' defined theoretically and practically by the US Environmental Protection Agency (USEPA) whose underlying reasoning has three parts: (i) Achieving a dose sufficiently high to kill all RR genotypes is unrealistic, so the high dose should instead kill almost all SR to ensure resistance remains recessive (the theoretical definition is that dominance is restricted to < 0.05). (ii) Dominance of resistance is difficult to anticipate, so the high dose must be defined practically: they chose a “high dose” as that which kills ≥ 99.99% of SS insects. (iii) A dose that kills such a high proportion of SS insects should kill enough SR genotypes to keep dominance below 0.05 and, in turn, slow the evolution of resistance. There is evidence that the high-dose approach works, Tabashnik & Carrière (2017) report that where resistance to Bt crops has evolved (17 cases), none met the high-dose standard, whereas those cases where resistance has not evolved (13 cases), nine met the high-dose standard. This high-dose approach is relevant to the deployment of new insecticides to which resistance has not yet arisen. Once resistance is observed then the new insecticides are already on their way to failure, so an obvious question is how resistance can be avoided in the first place. The logic must be to prevent a window of dominance from occurring, and the arguments above suggest this may be avoided by maintaining kill rates of the susceptibles ≥ 99.99%. Such high SS mortality is associated with low SR mortality in the tick data that we replot (Fig 5) but the Culex data (Fig 6) indicates that associated SR mortalities can be low. Whether maintaining 99.99% SS mortality is even operationally practical, given the decay of externally applied insecticides, is questionable but we would concur with the USEPA conclusion that if effectiveness against the susceptibles falls then it is likely that the SR genotypes will be surviving and that a window of dominance is open and operating.

**Appendix S5. How resistance encoded by single genes or by polygenes affect the impact of windows of selection**

Our considerations of windows of selection and dominance have assumed resistance is coded by a single gene. Windows of selection are also likely to apply when insecticide resistance is a polygenic trait, encoded by alleles at many genes, each with a small effect. When resistance is polygenic, populations are assumed to have a normal distribution of tolerance/resistance levels to insecticide concentrations (Fig S5.1A, Ffrench-constant, 2004). Differential mortality, within this normal distribution, will favour the survival of resistant genotypes, and drive the evolution of greater resistance. At the high insecticide concentrations that occur immediately after deployment, it is likely that all the polygenic genotypes will be killed by the insecticide.In this case selection for resistance within the normal distribution is absent, and the window of selection is shut (Fig S5.1B t1). As concentrations decrease, it is likely that more resistant (polygenic) genotypes will survive. This generates differential survival and selection within the normal distribution, driving the evolution of polygenic resistance; the window of selection is now open (Fig S5.1B t2). Finally, as concentrations decay to the level that all genotypes survive, differential selection within the distribution disappears and the window of selection shuts (Fig S5.1B t3). This creates a pattern of selection for polygenic resistance through windows of selection (Fig S5.1C) that is qualitatively similar to that shown for a single gene in Fig 1C. It is possible, in principle, to measure the differential mortality within the normal distribution during the open windows of selection. This would generate a “selection gradient” which can be used in standard quantitative genetic methodology to predict how quickly it drives resistance in terms of the per-generation change in mean resistance level. The theory is well known but, to our knowledge, has not yet been applied in this context.

**Figure S5.1**. Likely windows of selection when resistance is polygenic. A. Normal distribution of resistance phenotypes likely with polygenic resistance. B. Mortality at three time points after insecticide deployment, across the range of resistance levels. When the insecticide is deployed (t1) high insecticide concentrations kill all and there is no selection gradient. There is also no selection gradient when the insecticide concentration has declined close to 0 (t3). In between these extremes, a selection gradient is created by the difference in mortality across the resistance levels (t2). C. Resultant change in selection gradient over time. (For clarity we have restricted this to a very simple scenario with just 3 time points and a linear selection gradient, one can imagine more detailed scenarios which will also result in a window with elevated selection at intermediate times and concentrations). Thus, polygenic resistance is likely to create a window of selection that is conceptually similar to the one we have demonstrated for monogenic resistance.


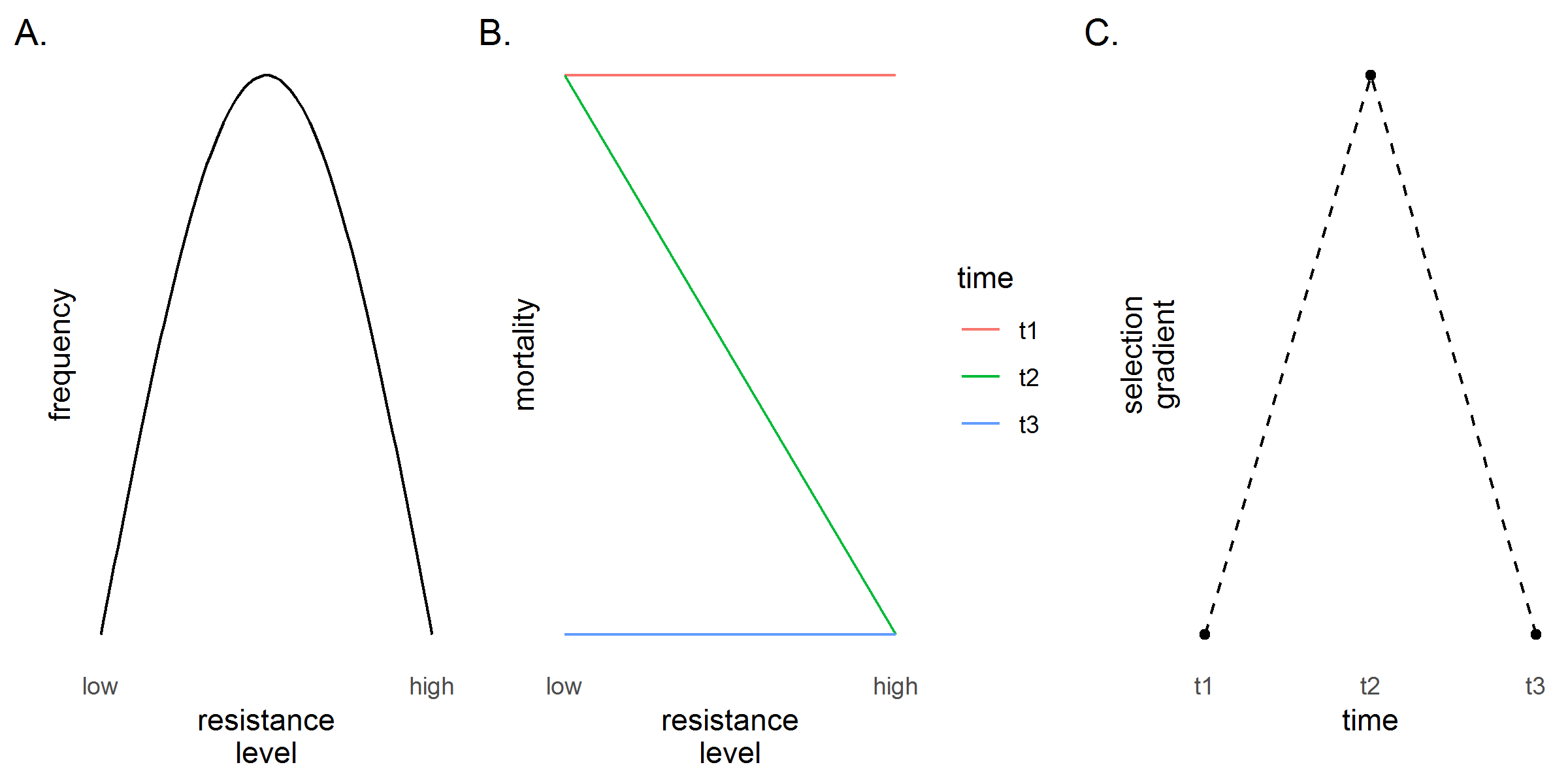

Supplement: Supplementary file 1 [file EVA-13-738-s001.docx]
